# The Candidate Photoperiod Gene *MtFE* Promotes Growth and Flowering in *Medicago truncatula*

**DOI:** 10.3389/fpls.2021.634091

**Published:** 2021-03-26

**Authors:** Geoffrey Thomson, Lulu Zhang, Jiangqi Wen, Kirankumar S. Mysore, Joanna Putterill

**Affiliations:** ^1^The Flowering Lab, School of Biological Sciences, University of Auckland, Auckland, New Zealand; ^2^Noble Research Institute, Ardmore, OK, United States

**Keywords:** *MtFE*, *MtFTa1*, *MtFTb*, NUCLEAR FACTOR-Y, CONSTANS, *Medicago truncatula*, *Arabidopsis thaliana*, photoperiodic flowering time

## Abstract

Flowering time influences the yield and productivity of legume crops. *Medicago truncatula* is a reference temperate legume that, like the winter annual *Arabidopsis thaliana*, shows accelerated flowering in response to vernalization (extended cold) and long-day (LD) photoperiods (VLD). However, unlike *A. thaliana*, *M. truncatula* appears to lack functional homologs of core flowering time regulators *CONSTANS* (*CO*) and *FLOWERING LOCUS C* (*FLC*) which act upstream of the mobile florigen FLOWERING LOCUS T (FT). *Medicago truncatula* has three LD-induced *FT-like* genes (*MtFTa1*, *MtFTb1*, and *MtFTb2*) with *MtFTa1* promoting *M. truncatula* flowering in response to VLD. Another photoperiodic regulator in *A. thaliana*, *FE*, acts to induce *FT* expression. It also regulates the FT transport pathway and is required for phloem development. Our study identifies a *M. truncatula FE* homolog *Medtr6g444980* (*MtFE*) which complements the late flowering *fe-1* mutant when expressed from the phloem-specific *SUCROSE-PROTON SYMPORTER 2* (*SUC2*) promoter. Analysis of two *M. truncatula Tnt1* insertional mutants indicate that *MtFE* promotes flowering in LD and VLD and growth in all conditions tested. Expression of *MtFTa1*, *MtFTb1*, and *MtFTb2* are reduced in *Mtfe* mutant (NF5076), correlating with its delayed flowering. The NF5076 mutant plants are much smaller than wild type indicating that *MtFE* is important for normal plant growth. The second mutant (NF18291) displays seedling lethality, like strong *fe* mutants. We searched for mutants in *MtFTb1* and *MtFTb2* identifying a *Mtftb2* knock out *Tnt1* mutant (NF20803). However, it did not flower significantly later than wild type. Previously, yeast-two-hybrid assays (Y2H) suggested that *Arabidopsis* FE interacted with CO and NUCLEAR FACTOR-Y (NF-Y)-like proteins to regulate *FT*. We found that MtFE interacts with CO and also *M. truncatula* NF-Y-like proteins in Y2H experiments. Our study indicates that despite the apparent absence of a functional *MtCO-like* gene, *M. truncatula FE* likely influences photoperiodic *FT* expression and flowering time in *M. truncatula via* a partially conserved mechanism with *A. thaliana*.

## Introduction

Flowering time plays a central role in optimizing the productivity and overall yield of many crops and was frequently selected for in crop domestication (Kantar et al., 2017). The Fabaceae family (legumes) contains a number of staple crops (e.g., bean, chickpea, lentil, peas, soybean) for which there is great potential for genetic improvement (Foyer et al., 2016). An understanding of the regulation of flowering time in this family is critical to help realize this potential and facilitate the breeding of more productive varieties. Many temperate legume species, such as the reference species garden pea (*Pisum sativum* L.) and *Medicago truncatula* Gaertn., accelerate their flowering in response to vernalization (V) and long-day photoperiod conditions (LD; Highkin, 1956; Clarkson and Russell, 1975). However, at a molecular level the pathways underpinning these responses are not fully understood (Weller and Ortega, 2015; Weller and Macknight, 2019). Core components of the pathways described in other species, such as *CONSTANS* (*CO*) and *FLOWERING LOCUS C*, appear to be missing in temperate legumes (Kim et al., 2013; Wong et al., 2014). However, progress to date has demonstrated a conserved role for phytochrome and circadian clock genes acting upstream of *FLOWERING LOCUS T* (*FT*)*-like* genes in the regulation of flowering time in legumes (Weller and Ortega, 2015; Jaudal et al., 2020; Weller and Macknight, 2019).

Photoperiodic flowering in eudicots is best characterized in *Arabidopsis thaliana* (L.) Heynh which incorporates light signaling, the circadian clock, and flowering time genes. The circadian clock acts through a complex of GIGANTEA (GI) and FLAVIN-BINDING, KELCH REPEAT, F-BOX 1 (FKF1) repressing expression of *CYCLING DOF FACTOR* (*CDF*) genes, encoding transcription factors which otherwise act to rhythmically repress the key transcription factor *CO*. The photoperiod pathway centers on *CO*, which is further regulated by photoreceptors like CRYPTOCHROME2 and PHYTOCHROME A (PHYA), which stabilize the CO protein at the end of LD (Andrés and Coupland, 2012; Song et al., 2015; Kinoshita and Richter, 2020). CO acts to directly induce the potent mobile floral signal encoded by *FT* (Turck et al., 2008). At least one CDF is also able to directly regulate *FT* (Song et al., 2012).

Like many species, homologs of *FT* have been identified in temperate legumes (Hecht et al., 2011; Laurie et al., 2011; Ortega et al., 2019). There are three distinct clades of *FT-like* genes in legumes (*FTa*, *FTb*, and *FTc*) and there are examples from all three clades regulating flowering time. The temperate legume reference species pea and *M. truncatula* possess representatives of all three (Hecht et al., 2011; Laurie et al., 2011). In these species, a *FTa* clade gene named *FTa1* integrates both LD photoperiod and V signals and is the predominant floral signal (Beveridge and Murfet, 1996; Hecht et al., 2011; Laurie et al., 2011; Jaudal et al., 2013, 2019; Yeoh et al., 2013). The function of the additional *FT-like* genes present in these species is currently unknown. Experimental evidence from pea is strongly suggestive that a secondary floral signal exists in this species with *PsFTb2* being a strong candidate, as it is quickly and strongly induced by LD conditions (Hecht et al., 2011). Consistent with this hypothesis the pair of *Medicago MtFTb* genes are also responsive to LD (but not V), suggestive of a role in floral activation (Laurie et al., 2011).

However, the functional role of a *CO-like* gene appears not to be conserved in the temperate legumes. For example, in pea the expression of *PsCOLa*, the most similar *CO-like* gene to *CO*, is not perturbed in circadian clock mutants (Hecht et al., 2007; Liew et al., 2009). In *M. truncatula*, mutants of the three *CO-like* genes most similar to *CO*, exhibit no difference in flowering time when vernalized plants were grown in LD (Wong et al., 2014). In the more distantly related tropical legume soybean [*Glycine max* (L.) Merr.] a pair of *CO-like* genes in soybean do act upstream of *FT-like* genes. However, they act to suppress, rather than activate, flowering and are therefore not functionally equivalent to *CO* in *A. thaliana* (Cao et al., 2015).

However, despite the apparent absence of a *CO-like* function, photoreceptors and the circadian clock play a significant role. In pea the circadian clock is transcriptionally upstream of *FT-like* genes (Hecht et al., 2011; Weller and Ortega, 2015; Weller and Macknight, 2019). Furthermore, the expression of *FT-like* genes are significantly reduced in *LATE1* (*Psgi*) mutants (Hecht et al., 2007, 2011) and a dominant late flowering pea *CDF* mutant was recently shown to act in the same pathway (Ridge et al., 2016). Similarly, in *M. truncatula* overexpression of the *MtCDFd1_1* gene resulted in late flowering relative to wild type plants in LD photoperiods resulting in the plants flowering in a day-neutral manner (Zhang et al., 2019). In addition, in both pea and *M. truncatula*, homologs of *PHYA* strongly promote flowering in LD with *Mtphya* mutants having strongly reduced levels of the LD-induced *FT-like* genes (Weller et al., 2001, 2004; Jaudal et al., 2020).

In soybean, the legume-specific B3 domain transcription factor *E1* has been shown to be an important regulator of *FT-like* genes. *E1* is photoperiod responsive and expressed in LD where the encoded protein acts to repress flowering (Xia et al., 2012; Xu et al., 2015). *E1-like* genes have been identified in temperate legumes like *M. truncatula* and *Mte1* mutants have a small delay in flowering (Zhang et al., 2016). We recently also demonstrated that *MtE1* expression is reduced in a *Mtphya* mutant (Jaudal et al., 2020). This shows similarities with *E1* in soybean where expression is completely lost in *e3e4* (*phya-like*) mutants (Xia et al., 2012; Lu et al., 2017). However heterologous expression of *MtE1* in soybean was unable to rescue the moderate early flowering phenotype of an *e1-as* leaky allele, as soybean *E1* can (Zhang et al., 2016). This, along with the small effect on flowering, suggest *MtE1* does not appear to play as central a role in the regulation of flowering in *M. truncatula* as *E1* does in soybean.

To shed further light on potential direct regulators of *FT-like* genes, and the undescribed functions of the *FT-like* genes themselves, a transcriptomic approach has previously been described to identify novel candidate *M. truncatula* photoperiod genes (Thomson et al., 2019). A complementary strategy is to screen candidate genes which have not previously been considered. One such candidate is *FE*, as the *fe-1* mutant has a late flowering phenotype in *A. thaliana* (Koornneef et al., 1991). *Arabidopsis FE* was recently cloned as a missense mutation in a gene encoding a phloem-specific SHAQKYF-class MYB-related protein known as ALTERED PHLOEM DEVELOPMENT (APL), which is essential for development of the vasculature (Bonke et al., 2003; Furuta et al., 2014; Abe et al., 2015). In *A. thaliana* FE/APL contributes to the transcriptional activation of *FT* in a LD-specific manner (Abe et al., 2015) and this is thought to occur in concert with a NUCLEAR FACTOR-Y (NF-Y) complex, which includes CO (Shibuta and Abe, 2017). Interestingly, in *A. thaliana* FE/APL also transcriptionally regulates components of the FT transport pathway including *FT-INTERACTING PROTEIN 1* (*FTIP1*) and *SODIUM POTASSIUM ROOT DEFECTIVE 1* (NaKR1; Abe et al., 2015; Shibuta and Abe, 2017). NF-Y complexes are known to regulate flowering in many species (Li et al., 2011; Kim et al., 2016; Zhao et al., 2016). It is thus conceivable that homologs of FE/APL may also play a role in the regulation of flowering in other species, such as those from the legume family.

Here we utilize the *M. truncatula Tnt1* insertional mutant population (Tadege et al., 2008; Cheng et al., 2014) to screen for mutants in potential upstream regulators of *FT-like* genes and in *FT-like* genes for which mutants have not previously been described. This resulted in the identification of a *Mtfe* mutant which has reduced growth and is later flowering in LD and vernalized LD (VLD) photoperiods with a reduction in expression of three LD-induced *FT-like* genes *MtFTa1*, *MtFtb1*, and *MtFTb2*. Molecular characterization also indicates that *MtFE* is broadly expressed and is able to complement the *fe-1* mutation in *A. thaliana*. MtFE interacts with CO and *M. truncatula* NF-Y-like proteins in yeast two-hybrid experiments. In addition, we identified a *Mtftb2* mutant, but this line flowers at a similar time to wild type plants.

## Materials and Methods

### Reference Genome

The *M. truncatula* genome sequence (version Mt4.0), generated from the accession “Jemalong A17,” was used as a reference for gene identifiers throughout the text (Young et al., 2011; Tang et al., 2014). In addition, the *M. truncatula* R108 genome assembly was used alongside to aid primer design (Moll et al., 2017). Primers were designed using the Primer3 (v. 2.3.4; Untergasser et al., 2012) plugin for the latest version of Geneious (≥v8.0; Kearse et al., 2012). Primer sequences are available in Supplementary Table S1. The TAIR10 genome assembly of *A. thaliana* was referred to when required (Berardini et al., 2015). The Tobacco DNA for retroviral-like transposon *Tnt1-94* sequence (GenBank ID: X13777) was used as the reference *Tnt1* retrotransposon sequence.

### Growth and Phenotyping of *Medicago truncatula* Plants

For *M. truncatula*, the wild type accession R108-1 (c3; referred to as R108; Hoffmann et al., 1997; Trinh et al., 1998) was used. Mutant *M. truncatula* lines were sourced from the *Tnt1* retrotransposon population maintained by the Noble Research Institute (Ardmore, OK, United States; Tadege et al., 2008). They were selected on the basis of the similarity of the gene of interest and associated sequence tags (FSTs) flanking reported *Tnt1* insertion events in the *M. truncatula* Mutant Database of *Tnt1* FSTs (Tadege et al., 2008; Cheng et al., 2014).

*Medicago truncatula* seeds were scarified by softly scraping them between two pieces of sandpaper (grade P160) and subsequently germinated in the dark in gently shaking tubes of water at 15°C for 24 h. Germinated seeds were then vernalized by being transferred to damp petri dishes and incubated at 4°C for 21 days. The seedlings were subsequently planted in seed raising mix (Daltons Ltd., New Zealand) in individual cell pots. Soil was kept moist with a complete liquid nutrient media (without Na_2_SiO_3_; Gibeaut et al., 1997). At 11 days plants were transplanted to 2 L pots containing potting mix (Daltons Limited, New Zealand) with added vermiculite and sand in a 9:3:1 ratio. Plants were watered twice a week, once with tap water and once with a complete liquid nutrient media (without Na_2_SiO_3_; Gibeaut et al., 1997).

Plants were grown at 22°C either in a controlled walk-in room in LD (16 h light/8 h dark) or in reach-in growth cabinets (Percival Scientific Incorporated, IA, United States^1^), predominantly for SD (8 h light/16 h dark). Light intensities were 120–150 μmol m^−2^ s^−1^. This was in accordance with Institutional Biological Safety Committee approval GMO08-UA006.

*Medicago truncatula* flowering time was scored as the number of days between sowing and the appearance of the first floral bud. At this time the total number of nodes on the primary axis of the plant was also recorded as a measure of developmental stage. Results were plotted with 95% confidence ellipses (2D analog of a confidence interval) to indicate variation within a population. Specific comparisons were made using a Welch two-sample *t*-test (*α* = 0.05) and when required one way ANOVA (type III sums of squares; *α* = 0.05).

*Medicago truncatula* plant heights were measured using a standard ruler with a millimeter level scale with the first node (where the monofoliate leaf diverges from the stem) is taken as point 0 mm. The ruler is then used to measure the distance of the nodes up the primary axis from this point. Results were plotted with 95% confidence intervals.

### Growth and Phenotyping of *Arabidopsis thaliana* Plants

This study also makes use of the *A. thaliana* Landsberg *erecta* (Ler-0) accession (Rédei, 1962) and the *fe-1* mutant. The *fe-1* mutant is a missense mutation in *ALTERED PHLOEM DEVELOPMENT* (At1G79430) generated by treating Ler-0 with ethyl methanesulphonate (Koornneef et al., 1991; Abe et al., 2015).

*Arabidopsis thaliana* seeds were submerged in sterile water and placed at 4°C in dark conditions for 2–3 days to overcome embryonic dormancy. Using a blunt toothpick *A. thaliana* seeds were then placed onto blocks of Grodan® Classic Rockwool soaked in a complete liquid nutrient media (without Na_2_SiO_3_; Gibeaut et al., 1997). They were placed in a grid accommodating up to 24 plants and grown in a controlled growth room at 22°C in LD conditions. The light intensity was 120 μmol m^−2^ s^−1^. This was in accordance with Institutional Biological Safety Committee approval GMO08-UA006.

Flowering time of *A. thaliana* was scored as the days between sowing and the appearance of the first floral bud. At this time the total number of rosette and cauline leaves present were also recorded. Statistically different results were assessed *via* a one way ANOVA (type III sums of squares; *α* = 0.05).

### Genotyping of Plants

Plants were genotyped using PCR with the Phire Hot Start II DNA Polymerase (Thermo Fisher Scientific Inc., MA, United States^2^) following the manufacturer’s instructions. This involved two reactions, the results of which could be used to infer the genotype of the plant. The first reaction used a gene-specific pair of primers which amplify a product spanning the insertion site of the *Tnt1* retrotransposon, as reported by the flanking sequence tag. If the *Tnt1* was present, this reaction should not amplify as any product would be too long (the *Tnt1* is 5.3 kb). The second reaction utilizes a pair of primers with one from the gene and one from the retrotransposon. If this reaction amplifies a product of appropriate size (estimated from the distance of the primer to the site of insertion), the *Tnt1* is present. If both reactions produce bands of the appropriate sizes the plant is inferred to be heterozygous for the insertion. If only one successfully amplifies then the plant is inferred to be homozygous for the product amplified. Primers used in this study are listed in Supplementary Table S1.

### Assaying Gene Expression *via* qRT-PCR

RNA from leaf tissue was extracted using the RNeasy Plant Mini Kit (Qiagen, Germany^3^) following the manufacturer’s instructions and 8 μg was then DNase treated with the TURBO DNA-free™ Kit (Invitrogen™, trademark of Thermo Fisher Scientific Inc.; MA, United States^2^). First-strand cDNA was then synthesized using 1 μg of DNase treated RNA. This was done using SuperScript™ IV Reverse Transcriptase (Invitrogen™, trademark of Thermo Fisher Scientific Inc.; MA, United States^2^) using the G775 primer as a Oligo(dT) primer following the manufacturer’s instructions.

The relative abundances of cDNA sequences in a sample, as a measure of gene expression, was assayed using real time quantitative PCR (qRT-PCR). This utilized SYBR™ Green PCR Master Mix Applied Biosystems™, trademark of Thermo Fisher Scientific Inc.; MA, United States^2^) and the QuantStudio 5 Real-Time PCR System (Applied Biosystems™, trademark of Thermo Fisher Scientific Inc.; MA, United States^2^). Analysis of the qRT-PCR data was enacted using the 2^−∆∆Ct^ algorithm (Livak and Schmittgen, 2001) and used *Medtr6g084690* which encodes a SERINE/THREONINE PROTEIN PHOSPHATASE 2A REGULATORY SUBUNIT (PP2A) as a reference gene (Kakar et al., 2008).

Each data point is from three independent biological replicates. Each replicate consisted of a pool of leaf tissue from either two or three independent plants (unless otherwise indicated). Statistically different gene expression measures were assessed from *post hoc* Tukey-adjusted comparisons of a linear model (*α* = 0.05). Primers used in this study are listed in Supplementary Table S1.

### *Arabidopsis thaliana* Transformation

Two constructs were made using the *A. thaliana SUCROSE-PROTON SYMPORTER 2* (*SUC2*) promoter in the pSAK778_SUC2 vector (Varkonyi-Gasic et al., 2013); *AtSUC2*::*Medtr6g444980* (*MtFE*) and *AtSUC2*::*AT1G79430* (*FE/APL*). Transgenic *A. thaliana* plants (both wild type L*er*-0 and *fe-1*) were generated using the floral dip method (Clough and Bent, 1998) utilizing the *A. tumefaciens* strain ‘GV3101’ (Koncz and Schell, 1986). Since the pSAK778_AtSUC2 vector confers resistance to kanamycin, T_1_ seeds were germinated on solid Murashige and Skoog media including vitamins with 100 μg/ml kanamycin. Plants successfully growing on the selective media after 12–15 days were then transferred to Grodan® Classic Rockwool soaked in a complete liquid nutrient media (without Na_2_SiO_3_; Gibeaut et al., 1997).

### Yeast-Two-Hybrid Assay

The coding sequences of the genes assayed by the Y2H method were commercially synthesized with flanking attB1/attB2 sites and cloned into both the pDEST™22 and pDEST™32 vectors using the Gateway® cloning system. These two vectors facilitate fusion of a gene of interest to the GAL4 DNA activation domain (AD) and GAL4 DNA binding domain (BD), respectively. Transformation of the haploid *Saccharomyces cerevisiae* Meyen ex E.C. Hansen strains P69-4a and P69-4α (James et al., 1996) was enacted using a lithium acetate method (Schiestl and Gietz, 1989) and grown on Synthetic Defined (SD) media [0.67% (w/v) Yeast nitrogen base without amino acids; 1.7% (w/v) Agar; 111 mM Glucose; 222 μM Adenine; 205 μM Lysine; 134 μM Methionine; 178.4 μM Uracil; 98 μM Tryptophan; 128.9 μM Histidine; 457.4 μM Leucine; pH 5.8]. Specifically, vectors with a pDEST™22 backbone were transformed into the PJ69-4a strain and plated on SD media lacking tryptophan (SD-W) and vectors with a pDEST™32 backbone were transformed into the PJ69-4α strain and plated onto SD media lacking leucine (SD-L). Diploid *S. cerevisiae* were generated *via* mating two haploid cells together in a 500 μl culture of Yeast-Extract Peptone Adenine Dextrose Media [1% (w/v) Yeast-extract; 2% (w/v) Peptone; 296 μM Adenine; 122 mM Glucose]. The culture was incubated at 28°C for 24 h while shaking at 180 rpm. It was then plated onto SD media lacking both leucine and tryptophan (SD-LW).

The Y2H assay was performed by suspending successfully mated diploid cells in 100 μl of water. This was performed in a 96-well plate so that a 96-pin applicator could be used to transfer the suspended cells onto SD media lacking leucine, tryptophan, and histidine (SD-LWH). The SD-LWH plates were done in triplicate as three technical replicates. The cells were incubated for 10 days at 28°C. This was repeated with the addition of 3-amino-1,2,4-triazole (3AT), which acts as a competitive inhibitor, at concentrations of 1, 2, 10, 25, 50, and 100 mM. Colonies were scored on a scale of 0 (no growth) to 3 (strong growth). Results were consistent across the technical replicates. In addition to the negative controls created by mating empty vectors, known positive and negative controls from the ProQuest™ Two-Hybrid System were also grown alongside (Thermo Fisher Scientific Inc.; MA, United States^2^).

## Results

### Identification of a *FE* Homolog in *Medicago truncatula*, *MtFE*

**Figure 1:**
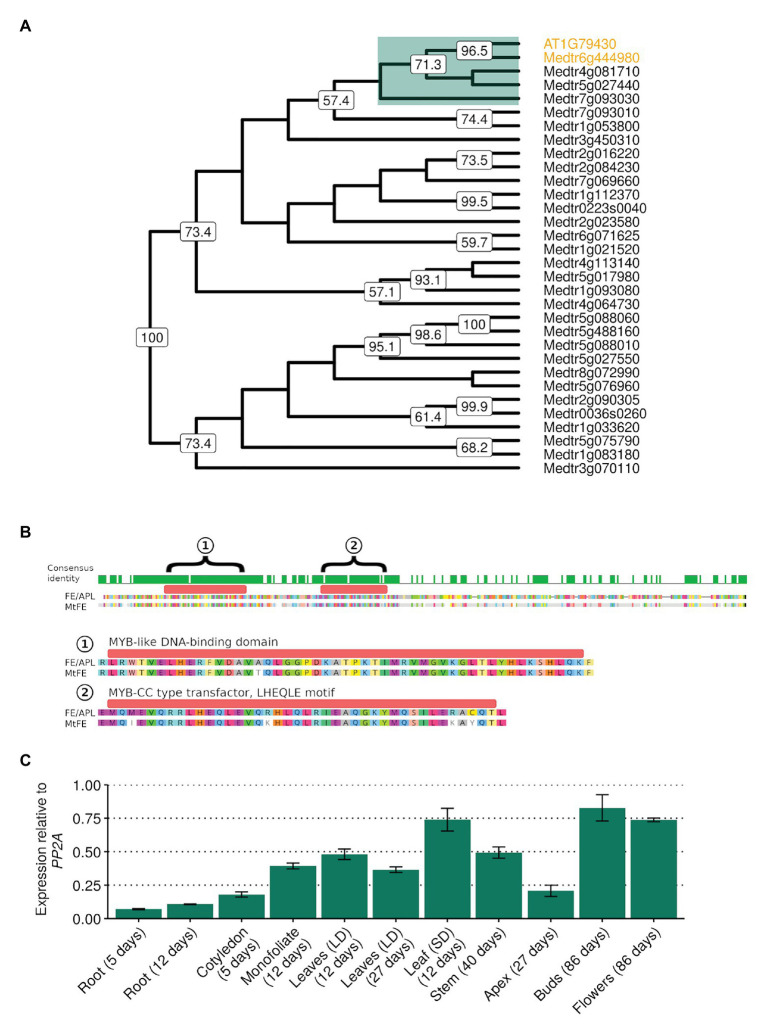
MtFE (Medtr6g444980) sequence similarity to *Arabidopsis thaliana* FE/APL (At1g79430) and the broad expression of *MtFE* in *Medicago truncatula* tissues. **(A)** is a neighbor-joining cladogram of *A. thaliana* FE/APL and *M. truncatula* SHAQYF class MYB-like proteins after alignment with MUSCLE (Edgar, 2004). Medtr6g444980 (MtFE) and At1g79430 (AtFE/APL) are highlighted in orange. They fall into a subclade of five proteins (highlighted in green) that possess both a MYB-like DNA-binding domain (BD) and a MYB-CC type transfector, LHEQLE motif. Node numbers reflect support from 1,000 bootstraps. **(B)** is an alignment of MtFE to AtFE/APL. The predicted proteins are 60% identical. The green histogram above the sequence indicates areas of identical sequence with the two domains predicted by the Pfam database (https://pfam.xfam.org/) annotated and magnified below; (1) a MYB-like DNA-BD and (2) a MYB-CC type transfector, LHEQLE motif. **(C)** is the mean relative expression using qRT-PCR of *MtFE* in three independent biological replicates of different tissues harvested 2 h after dawn (ZT2) grown in long-day photoperiods (LD) unless otherwise shown (short-day photoperiods, SD). Expression is relative to the reference gene *SERINE/THREONINE PROTEIN PHOSPHATASE 2A REGULATORY SUBUNIT A* (*PP2A*) with error bars representing standard errors.

In order to investigate whether a *FE* homolog in *M. truncatula* might play a role in flowering time, reciprocal BLAST searches using FE/APL as a query (Altschul et al., 1990) were used to identify *Medtr6g444980*. In a neighbor-joining tree of the 31 SHAQKYF-class MYB-like putative transcription factors identified in the *M. truncatula* genome, Medtr6g444980 clades closest to FE with strong bootstrap support (Figure 1A). *MtFE* encodes a protein 54.3% identical to FE/APL (Figure 1B) and contains a predicted MYB-like DNA-BD of the SHAQKYF-class of MYB-related proteins. FE and Medtr6g444980 also both possess a MYB-CC type transfector LHEQLE motif, the function of which is unknown. Only three other *M. truncatula* SHAQKYF-class MYB-like proteins have MYB-CC type transfector LHEQLE motifs (Medtr4g081710, Medtr5g027440, Medtr7g093030) and these proteins are only 32–36% identical to FE/APL. We thus named *Medtr6g444980* as *MtFE*.

The expression of *MtFE* in a range of different *M. truncatula* tissue types, including roots, leaves, and flowers was then assessed by qRT-PCR. It was observed that *MtFE* is broadly expressed in all the tissues assayed (Figure 1C), consistent with published transcriptome datasets (Benedito et al., 2008; Krishnakumar et al., 2015).

### The *AtSUC2*::*MtFE* Transgene Complements the *Arabidopsis thaliana fe-1* Mutant

To test if *MtFE* can complement the late flowering *A. thaliana fe-1* mutant, *MtFE* was transformed into the mutant under control of the phloem specific SUCROSE-PROTON SYMPORTER 2 (*SUC2*) promoter. This promoter was used to provide strong expression in the phloem companion cells which is the tissue where FE/APL regulates *FT* and FT transport (Shibuta and Abe, 2017). An *A. thaliana SUC2*::*AtFE* construct was made as a positive control. Both constructs were also transformed into the L*er*-0 background to test if overexpression further accelerates flowering.

**Figure 2:**
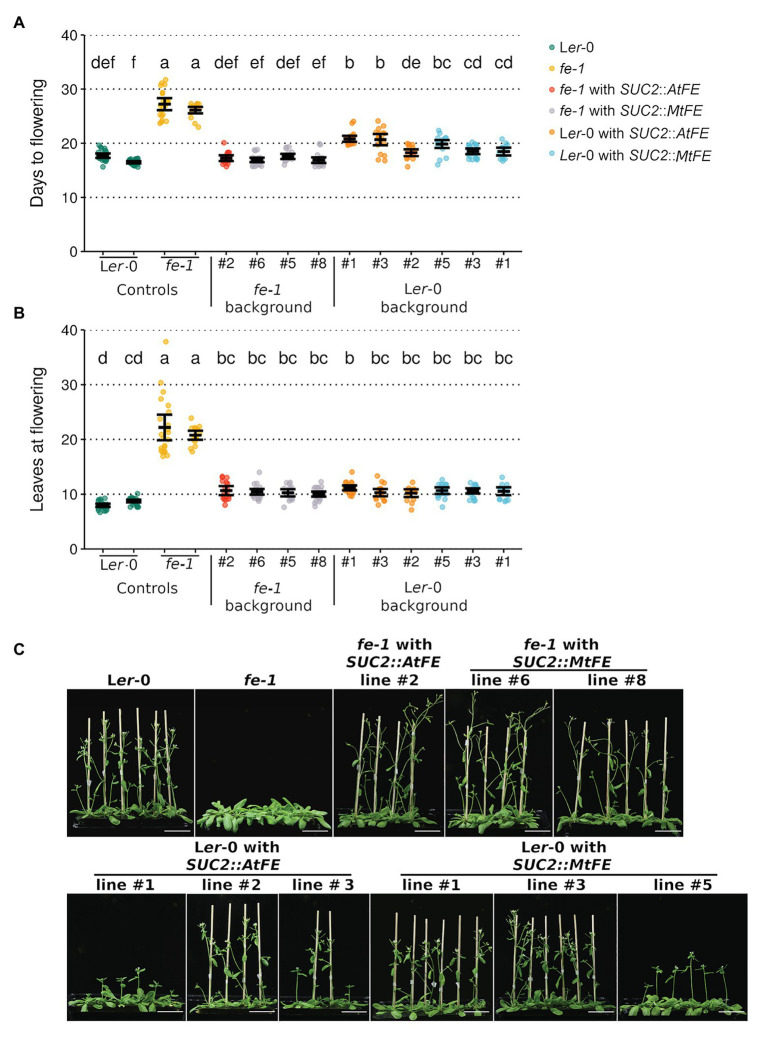
*MtFE* complements the *A. thaliana fe-1* mutant. **(A)** and **(B)** plot the flowering time of T_3_ *SUC2::MtFE* and *SUC2::AtFE/APL* plants in both the *fe-1* and L*er*-0 backgrounds. Flowering time was measured in **(A)** days and **(B)** total leaves at the time of flowering. Individual plants (*n* = 15–24) are plotted as points and black bars depict the mean bounded by 95% confidence intervals. Different letters indicate significantly different results *via* a one-way ANOVA (type III sums of squares; *α* = 0.05). **(C)** presents representative photographs of 27 days old plants. The white scale bar represents 5 cm.

Three representative lines from each of the *fe-1 SUC2*::Mt*FE*, L*er*-0 *SUC2*::At*FE* and L*er*-0 *SUC2*::Mt*FE* experiments along with one *fe-1 SUC2*::At*FE* line were taken to the T_3_ generation, and their flowering time scored (Figure 2). It was found that *SUC2*::*MtFE* and *SUC2::AtFE* can both complement the *fe-1* mutant phenotype to return to wild type-like flowering time (Figure 2). This indicates that MtFE may share a similar biochemical function with AtFE, in the *A. thaliana* system. However, expression of either transgene from the *SUC2* promoter in the wild type L*er*-0 did not appear to further accelerate flowering, but in some cases, a slight delay in flowering was observed (Figure 2).

### The NF5076 *Mtfe* Mutant Has Reduced Growth and Delayed Flowering in LD and VLD Conditions Compared to Wild Type Plants

**Figure 3:**
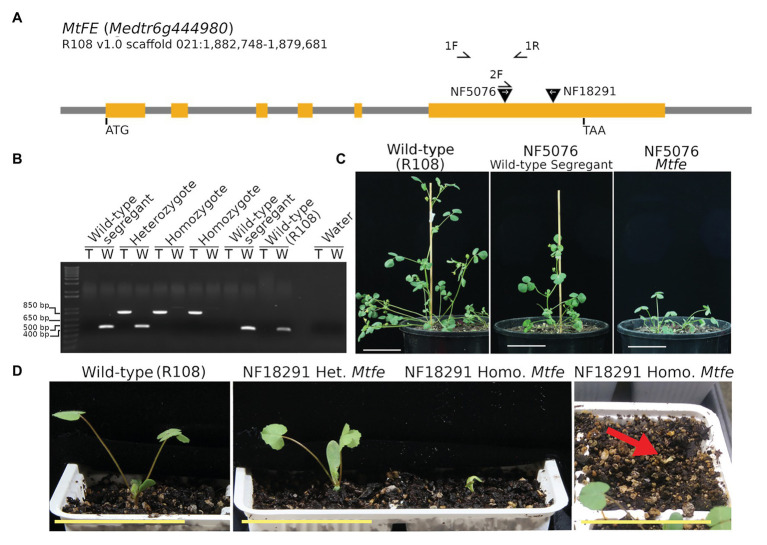
*Medicago truncatula MtFE* mutant NF5076 has a condensed aerial architecture while NF18291 exhibits seedling lethality. **(A)** is a schematic of the *MtFE* locus with the orange rectangles representing the exons and two inverted black triangles the location of the confirmed *Tnt1* insertions. The white arrows denote the orientation of the *Tnt1* insertion. Primers used are indicated by half arrows. **(B)** is an example of a genotyping gel for the NF5076 line with two reactions per plant. The first amplifies the *Tnt1* insertion allele (T) with primers 2F and 1R (854 bp) and the second amplifies the wild type R108 allele (W) with primers 1F and 1R (416 bp). **(C)** are photos of 41 days old NF5076 plants which have a condensed architecture phenotype relative to the wild type R108. **(D)** shows the small seedling growth phenotype of NF18291 *Mtfe* plants at 10 days old (left and middle panel). The far-right panel shows how at 14 days these plants atrophy and die. All plants were grown in VLD. All scale bars are 5 cm.

To identify *Mtfe* mutants, the genomic *MtFE* sequence was used as a query to search the *M. truncatula* Mutant Database of *Tnt1* FSTs (Tadege et al., 2008; Cheng et al., 2014). Six *Tnt1* lines with reported insertions in the *MtFE* locus were screened. *Tnt1* insertions were confirmed in two lines, NF5076 and NF18291, both in the sixth exon downstream of the predicted MYB-like DNA-BD and MYB-CC type transfector LHEQLE motif (Figures 3A,B; Supplementary Table S2).

We observed that the NF5076 *Mtfe* homozygous plants were compromised in their growth, being much smaller and more compact than their wild type R108 counterparts in VLD (Figure 3C). NF5076 wild type segregants were also smaller relative to the wild type R108. However, these wild-type segregants were not nearly as small as the homozygotes (Figure 3C). Further analysis of the progeny of wild type segregants from NF5076 indicates that this small phenotype appears to be a heritable feature of the line (Supplementary Figure S1). The NF5076 segregating line also exhibited other phenotypes that did not segregate with the *Tnt1* insertion in *MtFE*. These phenotypes made propagation difficult. Several plants produced seeds which had not developed properly and presented as dark and small; these seeds did not germinate. Some plants also produced malformed barrels where the normal coiling of the barrel did not occur, and which did not produce viable seed (Supplementary Figure S1C).

**Figure 4:**
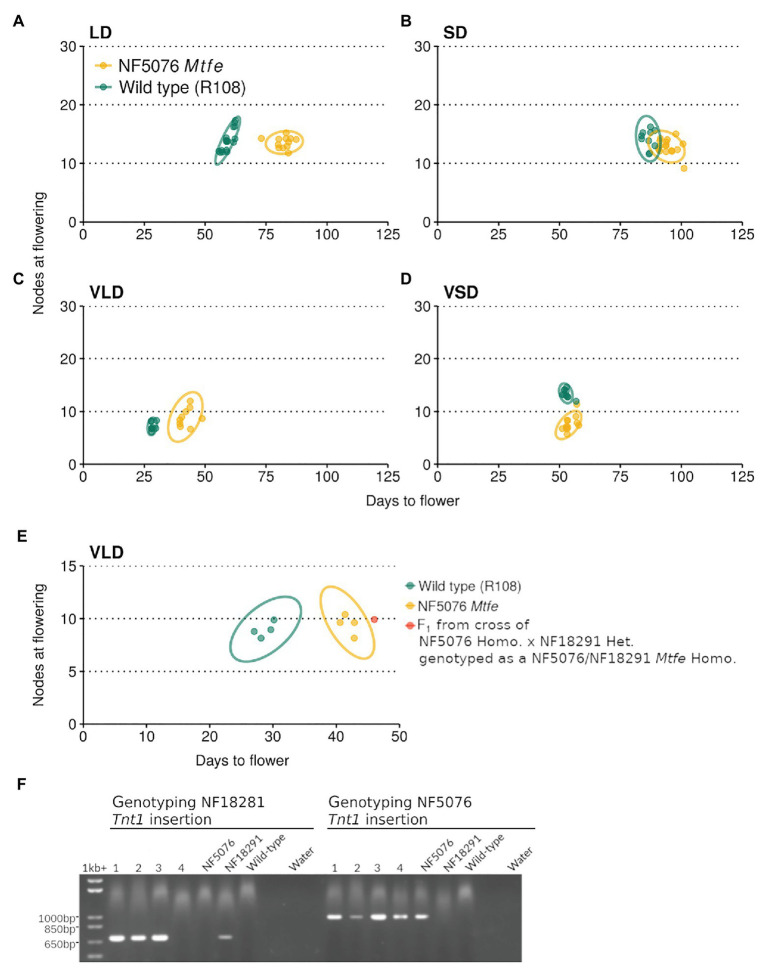
NF5076 *Mtfe* mutants have delayed flowering relative to wild type in days when grown in LD and VLD. The flowering time of *M. truncatula* NF5076 *Mtfe* mutants (*n* = 7–12) grown in **(A)** LD, **(B)** SD, **(C)** VLD, and **(D)** VSD are plotted relative to the wild type R108. **(E)** plots the flowering time in VLD of a NF5076 *Mtfe* x NF18291 *Mtfe* F_1_ plant relative to NF5076 *Mtfe* and wild type R108 plants. Note that heterozygous plants from both mutant lines flower at a similar time to wild type R108 (Supplementary Figures S2, S4). **(F)** Genotyping of the four F1 progeny from a cross of homozygous NF5076 *Mtfe* mutants with a heterozygous NF18291 plant, showing that three of the F1 plants (lanes 1–3) are bi-allelic mutants. Of these three plants, one survived and two died prior to flowering. Genotyping of the NF18291 insertion utilized the Tnt1_R/Medtr6g444980_2127_R primer pair which specifically amplifies a 760 bp product. Genotyping the NF5076 insert used the Tnt1_F/Medtr6g444980_2127_R primer pair with a 1,001 bp product. Flowering time graphs are plots of days to flower against nodes on the primary axis at the time of flowering. Each point represents an individual plant. Variation is indicated by 95% confidence ellipses (2D analog of a confidence interval).

Next, NF5076 *Mtfe* homozygous plants were grown in contrasting photoperiod conditions (LD or short days, SD, with or without V). These plants had delayed flowering in both LD and VLD conditions relative to the wild type R108 (Figures 4A-D). In LD, NF5076 *Mtfe* plants flowered on average 22.7 days (38.2%) later than the wild type R108. Similarly in VLD, the NF5076 *Mtfe* homozygotes flowered 14.2 days later (50%) than the wild type R108. Interestingly, when grown in SD or VSD no difference in the flowering time of NF5076 *Mtfe* plants was observed relative to the wild type R108 (Figures 4B,D) but the small phenotype was also observed. These results overall suggest that *MtFE* appears to promote LD and VLD photoperiodic flowering, but also regulates normal plant growth.

The NF5076 *Mtfe* mutant was then backcrossed to the wild type R108 and a F_2_ population of 180 plants was grown in VLD (Supplementary Figures S2A,B). Taking 33 days as the cut-off for wild type flowering (on the basis of Figure 4C), the flowering time of the *Mtfe* homozygous segregants was, on average, significantly later than wild type R108 (ANOVA: *p* = 2.2 × 10^−16^; *α* = 0.05; Supplementary Figures S2A,B). These *Mtfe* homozygous segregants flowered late to similar degree to the previous grown NF5076 *Mtfe* plants grown in VLD (Figure 4C). In the segregating population as a whole, 119 plants flowered like wild type and 61 plants had delayed flowering. This deviates from the expected 3:1 ratio of a recessive pattern of inheritance (*χ*^2^: 7.59, *p* = 0.0059) with more late flowering plants than expected. Genotyping also differed from the expected segregation 1:2:1 ratio (*χ*^2^: 7.24, *p* = 0.027). We obtained a ratio of 55:72:53 (*MtFE*/*MtFE*: *MtFE*/*mtfe*: *mtfe*/*mtfe*), but the population was not biased towards homozygotes or wild type segregants. These discrepancies may be attributable to growth phenotypes in the line causing irregular patterns of inheritance. As shown in Supplementary Figure S2C, the mean of the distribution for the NF5076 line is significantly greater than that of the wild type. However, since there was a modest level of overlap in the flowering time distributions between the genotypes in the population, 100% co-segregation between late-flowering and NF5076 *Mtfe* homozygotes was not observed. Regardless, the NF5076 *Mtfe* insertion is strongly linked to the late-flowering phenotype (*χ*^2^: 104, *p* = 2.2 × 10^−16^). While it remains possible that another closely linked disrupted gene contributes to the late-flowering phenotype observed, it is more likely that the moderate delay to flowering is conferred by the *Mtfe* mutation in NF5076, rather than another closely linked mutation.

In addition to the days required to flower, the flowering time in *M. truncatula* is also measured by the number of nodes on the primary axis at the time of flowering. We observed that the difference in nodes between NF5076 *Mtfe* homozygous and wild type R108 plants was more subtle than the delay in days to flowering seen in the mutant. Nevertheless, in the populations grown in contrasting photoperiod conditions, plants grown in VLD had a small, but significant, difference with the NF5076 plants having 1.78 nodes more on average (Welch two-sample *t*-test, *p* = 0.016). In LD, plants had a similar number of nodes (13–14) as wild type R108. The difference in nodes in VLD was also seen in the larger F_2_ NF5076 *Mtfe* segregating mutant population where an increase of 1.75 nodes was observed relative to the wild type R108 plants grown alongside (ANOVA: *p* = 2.14 × 10^−5^; *α* = 0.05). The discrepancy in magnitude between the delay in days required to flower and nodes on the primary axis at the time of flowering, suggest that the flowering time phenotype in the NF5076 *Mtfe* mutant may, in part, be a consequence of an overall delayed rate of growth.

Plant height and primary axis node number were also measured in plants of the backcrossed F_2_ NF5076 *Mtfe* mutant population in VLD over a time course (33, 48, and 63 days; Supplementary Figure S3). We observed that the reduced growth phenotype of NF5076 *Mtfe* homozygous plants persists through development. Thus, even at the time of flowering, mutant plants did not resemble flowering wild-type R108 plants. Instead, they remained small and compact (Supplementary Figure S3A).

### The NF18291 *Mtfe* Homozygous Mutant Has a Seedling Lethal Phenotype

Unlike NF5076, *Mtfe* homozygotes from the second *Tnt1* insertion line, NF18291, could not be scored for flowering and growth because seedlings died within 14 days after planting (Figure 3D). Thus in the NF18291 line, the *Tnt1* insertion in *MtFE* either causes, or is closely linked, to a locus which causes seedling lethality. While both NF5076 and NF18291 *Tnt1* insertions occur in the sixth exon, they are in opposite orientations thus the consequences on *FE* expression and function may differ between the two lines.

To test for allelism between the two mutant lines, a plant heterozygous for the NF18291 *Mtfe* allele was crossed to a NF5076 *Mtfe* homozygote. Heterozygous plants from both these lines flower at a comparable time to wild type (Supplementary Figures S2, S4). Three F1 plants were bi-allelic mutants (Figure 4F). Two died before flowering, while the third plant had delayed flowering in days, like that of NF5076 *Mtfe* plants, with a similar number of nodes as the wild type R108 (Figure 4E). While the sample number is small, this suggests that NF18291 is allelic with NF5076 with regard to causing the seedling lethal phenotype. This is also consistent with the loss of function *fe/apl* mutation in *A. thaliana* being seedling lethal (Bonke et al., 2003). Unfortunately, flowering time was only able to be measured in the one surviving bi-allelic plant. This plant flowered late. This also is consistent with the two *Tnt1* insertions being allelic and suggests that disruption of *MtFE* in NF5076 causes the observed delay in days to flowering.

### The Expression of LD-Induced *MtFT* Genes and *FTIP1* and *NaKR1-like* Are Reduced in the NF5076 *Mtfe* Homozygous Mutant

**Figure 5:**
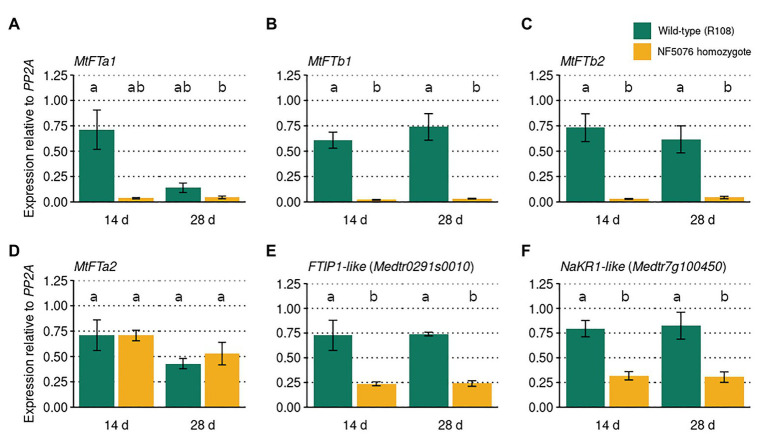
*MtFE* acts upstream of LD induced *FT-like* genes. The mean expression (*n* = three independent biological replicates) of **(A)** *MtFTa1*, **(B)** *MtFTb1*, **(C)** *MtFTb2*, **(D)** *MtFTa2*, **(E)** *FTIP1-like* (*Medtr0291s0010*), and **(F)** *NaKR1-like* (*Medtr7g100450*) in *M. truncatula* NF5076 *Mtfe* plants at two different ages (14 and 26 days old). Expression is relative to the housekeeper *PP2A*. All plants were grown in VLD and error bars depict standard errors. Different letters indicate significantly different results from *post hoc* Tukey-adjusted comparisons of a linear model (*α* = 0.05).

In *A. thaliana*, *FE/APL* regulates flowering time through the induction of *FT* (Abe et al., 2015). To investigate whether *MtFE* acts upstream of *FT-like* genes in *M. truncatula*, qRT-PCR was used to assay these genes in wild-type R108 plants and the NF5076 *Mtfe* mutant. Plants were grown in VLD and sampled at two timepoints, 14 and 28 days old. It was observed that the expression of *MtFTa1*, *MtFTb1*, and *MtFTb2* were consistently reduced relative to wild type R108 controls, but not *MtFTa2* (Figures 5A-D). This indicates that, similar to *FE/APL* in *A. thaliana*, *MtFE* also acts upstream of LD-induced *FT-like* genes in *M. truncatula*.

In *A. thaliana FE/APL* also regulates *FTIP1* and *NaKR1* (Abe et al., 2015; Shibuta and Abe, 2017). Thus the expression of *MtFTIP1* (*Medtr0291s0010*) and the *NaKR1-like* gene *Medtr7g100450* in *NF5076 Mtfe* plants was also assayed. Modest, but consistent, reductions in the expression of *MtFTIP1* relative to wild type were observed (Figure 5E). Consistent reductions in *Medtr7g100450* expression were also observed (Figure 5F). These results suggest that *MtFE* may also act upstream of these genes in *M. truncatula*.

The *M. truncatula* Mutant Database of *Tnt1* FSTs was also used to search for *Tnt1* lines with insertions in *MtFTIP1*, which encodes a protein 80% identical to FTIP1. Of the two lines identified (Supplementary Table S2), one line, NF10483, was confirmed to contain the reported *Tnt1* insertion 313 bp into the single exon gene in the forward orientation (Supplementary Figure S5A). However, the expression of *MtFTIP1* transcript downstream of the insertion was still detected in this line when assayed by qRT-PCR, albeit reduced relative to wild type R108 (Supplementary Figure S5B). Subsequently, a transcript extending from the *Tnt1* and into the gene was able to be amplified in both VLD and VSD. There is no open reading frame which spans the long terminal repeat of the *Tnt1*, but following the insertion, four of the first five open reading frames are in frame with *MtFTIP1* (including the first) and encode potential proteins 77–83% of the full-length MtFTIP1 protein. NF10483 plants homozygous for the *Mtftip1 Tnt1* insertion flowered at the same time as wild type R108 (Supplementary Figure S6). When grown in VLD the line flowered marginally earlier than wild type R108, regardless of the *Tnt1* insertion in *MtFTIP1* (Supplementary Figure S6E).

### MtFE Appears to Interact With NF-Y Complex Components in Yeast-Two-Hybrid Assays

Given that *MtFE* appears to act upstream of *FT-like* genes, this raises the question of how this mechanistically occurs. In *A. thaliana*, FE/APL may participate in the NF-CO complex regulating the expression of *FT* (Shibuta and Abe, 2017). While *M. truncatula* does not appear to possess a *CO-like* gene acting upstream of the *FT-like* genes (Wong et al., 2014), we hypothesized that MtFE might interact in a similar complex.

**Figure 6:**
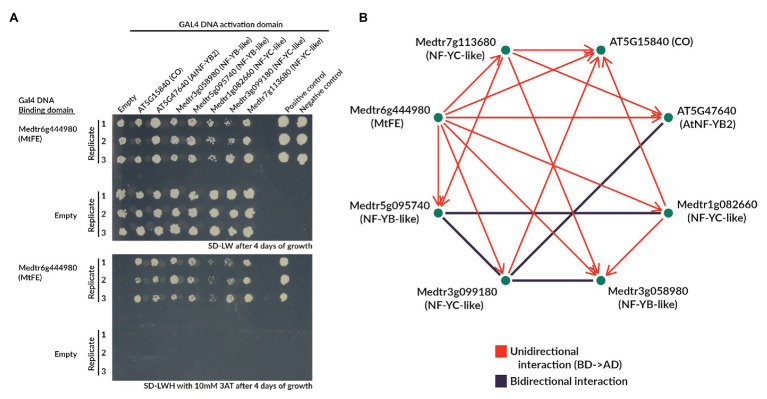
MtFE interacts with NF-Y-like proteins in yeast two-hybrid assays. **(A)** is an example of the yeast growth observed in an Y2H assay when MtFE was fused to the GAL4 DNA BD, and CO and NF-Y-like proteins were fused to the GAL4 activation domain (AD). Two photographs are shown, one on SD-LW media (upper panel), and the other on SD-LWH + 2 mM 3AT media (lower panel) selecting for yeast two-hybrid interactions. All assays were plated in triplicate. Also included are empty vector controls. **(B)** is a network summarizing the total observed interactions after 10 days of growth at 10 mM 3AT, excluding CO fused to the BD which showed auto-activation. Interactions only observed in one configuration of the GAL4 domains are in red and arrows extend from the protein fused to the BD to the protein fused to the AD. Bidirectional interactions (either configuration of GAL4 domains) are depicted in black.

To test this we selected five *M. truncatula* NF-Y proteins (two NF-YB-like and three NF-YC-like) based on sequence similarity to the NF-Y proteins demonstrated to regulate flowering time in *A. thaliana* (Supplementary Figure S7; Kumimoto et al., 2008, 2010). We then performed a Y2H assay with MtFE, CO, and AtNFY-B2 from *A. thaliana*, along with the five *M. truncatula* NF-Y proteins (Figure 6; Supplementary Figure S8).

Strikingly, when fused to the GAL4 DNA BD MtFE appeared to interact with all five *M. truncatula* NF-Y-like proteins (Figure 6A). MtFE also appeared to interact with AtNFY-B2 and CO. It did not interact with the empty vector control containing just the GAL4 DNA AD (Figure 6A). When MtFE was fused to the GAL4 DNA AD, it did not interact with any assayed proteins (Supplementary Figure S8). We also observed that the *M. truncatula* NF-YC-like protein Medtr3g099180 interacted in both directions with two *M. truncatula* NF-YB-like proteins Medtr3g058980 and Medtr5g095740, as well as At-NF-YB2 (Figure 6B; Supplementary Figure S8). A second NF-YC-like protein, Medtr1g082660, also interacted with the NF-YB-like Medtr5g095740 in both directions (Figure 6B; Supplementary Figure S8). Auto-activation was observed in all reactions where CO was fused to the GAL4 DNA BD so all interactions which involved this construct were excluded (Supplementary Figure S8). Auto-activation was not observed with any other construct (Supplementary Figure S8).

A network summarizing the total observed interactions is presented in Figure 6B. It shows all interactions observed after 10 days of growth at 10 mM 3AT, excluding CO fused to the GAL4 DNA BD which showed auto-activation.

### *MtFTb2* Does Not Have a Non-redundant Role in Flowering Time

Given that the NF5076 *MtFE* insertion strongly reduces the expression of the LD-induced *FT-like* genes we searched for *Tnt1* lines disrupting three *FT-like* genes for which mutants have not yet been analyzed. A total of nine lines were selected on the basis of reported insertions in the *MtFTa2* (three lines), *MtFTb1* (*Medtr7g006630*; four lines), *MtFTb2* (*Medtr7g006690*; two lines) loci (Supplementary Table S2).

**Figure 7:**
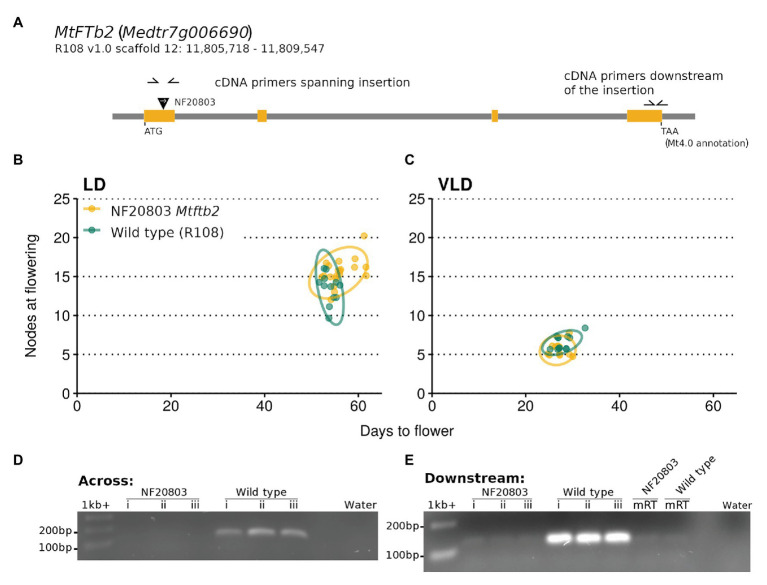
The NF20803 *Mtftb2* mutant flowers at a similar time to wild type R108. **(A)** is a schematic of the *MtFTb2* (Medtr7g006690) locus with the exons depicted in orange. The inverted black triangle indicates the location of the NF20803 *Tnt1* insertion and the white arrow denotes the orientation of the insertion. Primers used are indicated as half arrows. The flowering time of *M. truncatula* NF20803 *Mtftb2* mutants (*n* = 11–20) grown in **(B)** LD and **(C)** VLD are plotted below. Flowering time graphs are plots of days to flower against nodes on the primary axis at the time of flowering. Each point represents an individual plant. Variation is indicated by 95% confidence ellipses. Below are gels that depict the lack of amplification of the *MtFTb* gene in cDNA from the mutant compared to wild type R108 plants. Reactions in **(D)** used primers which span the *Tnt1* insertion and **(E)** used primers which are downstream of the insertion.

The predicted insertion was found in one of the two *MtFTb2* lines, NF20803. This line had an insertion in the first exon of the gene (Figure 7A). Growth of plants homozygous for this insertion did not differ in flowering time from the wild type R108 in VLD. However a slight trend to lateness was seen in LD, but this was not significantly different from wild type R108 (Figures 7B,C). PCR across the *Tnt1* insertion site using cDNA as the template did not amplify the transcript in the mutant, indicating that the insertion disrupted the mRNA transcript (Figure 7D). In addition, PCR of the *MtFTb2* transcript downstream of the insertion in NF20803 *Mtftb2* plants also did not amplify (Figure 7E). These results suggest that the loss of *Mtfb2* expression does not appreciably alter flowering time. Thus if it does regulate flowering time, it does so in a redundant manner.

In the remaining lines, the reported *Tnt1* insertions were confirmed in two of the three *MtFTa2* lines, NF9421 and NF19514. Homozygous mutant plants were grown in VLD, VSD, and SD where no difference in flowering time relative to the wild type R108 was observed (Supplementary Figures S9A-F). However the NF9421 line as a whole, regardless of the *Tnt1* insertion in *MtFTa2*, flowered marginally earlier than wild type R108 when grown in VSD (Supplementary Figure S9G).

Finally, of the three lines screened for reported *Tnt1* insertions in the *MtFTb1* locus, no lines were identified with the predicted insertions.

## Discussion

The constituents of the *M. truncatula* photoperiodic pathway which act directly upstream of *FT-like* genes are not currently known. This is because *CO-like* genes do not appear to function to promote flowering in the temperate legumes pea and *M. truncatula*, as *CO* does in *A. thaliana*. We have identified one candidate component, *MtFE*, and provided further insight into *FT-like* genes. To our knowledge, homologs of *FE/APL* have not been studied outside of *A. thaliana* prior to this study. The work presented here suggests that, like *A. thaliana FE/APL*, *MtFE* functions as a floral activator in LD and VLD photoperiods and can complement the late-flowering *A. thaliana fe-1* mutant. Furthermore, again like *FE/APL* in *A. thaliana*, *MtFE* is important for normal growth in *M. truncatula*.

The NF5076 *Mtfe* mutants are impaired in their growth and present with a compact aerial architecture. Given the role FE/APL plays in callose deposition and phloem development in *A. thaliana* (Bonke et al., 2003), it is possible *MtFE* also plays a role in these processes. Consistent with a broader function, the second *Mtfe* mutant identified, line NF18291, had a seedling lethal phenotype. This is similar to *A. thaliana fe/apl* knockout mutants which are seedling lethal too (Bonke et al., 2003). The reason for the difference in severity of phenotype between the NF5076 and NF18291 *Tnt1* insertions in exon six remains to be uncovered. However, they are inserted in opposite orientations. Thus the differing phenotypes may be due to the expression of different *FE*-related transcripts in each line. For example, we observed transcripts arising downstream from the NF10483 *Tnt1* insertion in *MtFTIP1*. We attempted similar assays of *FE* transcripts upstream and downstream of the insertions in the two *FE* lines. However, these were limited by the small amount of plant material we recovered for NF18291 (as the plants died very early).

In terms of flowering time, NF5076 *Mtfe* plants flowered in days on average 38% later than the wild type R108 in LD and 50% later in VLD. However, an increase in primary stem nodes at the time of flowering compared to wild type was only observed in the mutant in VLD. While a substantial cause of the delayed flowering may be the reduced rate of growth in the mutant, the reduction in *MtFTa1*, *MtFTb1*, and *MtFTb2* expression indicates that *MtFE* may also participate in the flowering time pathway. Intriguingly, despite *MtFE* being expressed in both SD and LD conditions, the delay in days required to flower in NF5076 *Mtfe* plants compared to wild type R108 appears only in LD and VLD.

Candidate upstream photoperiodic pathway components that are linked to FE/APL function in *A. thaliana* include the *NF-Y-like* genes. This highly conserved family of transcription factors is involved in the regulation of many developmental and stress responses across eukaryotes (Zhao et al., 2016). In *M. truncatula* they have previously been shown to participate in root nodulation (Laporte et al., 2014; Baudin et al., 2015; Rey et al., 2016). In our Y2H analysis, we show that these genes encode proteins which potentially interact with MtFE. These results are consistent with the role their homologs play in flowering time regulation in *A. thaliana* (Kumimoto et al., 2008, 2010). Thus *NF-Y-like* genes should be investigated as potential flowering time genes in *M. truncatula*. In addition, in Y2H assays MtFE and NF-YC-like proteins interact with AtCO. While *M. truncatula* appears to lack a functional *CO-like* homolog (Wong et al., 2014), other proteins containing the CCT domain regulate *FT-like* genes in many species (Ballerini and Kramer, 2011). This could potentially be the case in *M. truncatula* too (Thomson et al., 2019).

Other candidates which may act with MtFE to regulate flowering time could include NAC transcription factors, as in *A. thaliana FE/APL* regulates phloem development *via* a redundant pair of NAC transcription factors (Furuta et al., 2014). NAC transcription factors, in concert with a jumonji demethylase, have been demonstrated to repress *FT* and other flowering integrators (Ning et al., 2015). Given the ability of *FE/APL* to regulate NAC transcription factors in another context (*FE/APL* is also hypothesized to interact with jumonji demethylases; Abe et al., 2015) it is possible that *FE/APL* regulates these NAC transcription factors which influence flowering time. Although in this case repression of these transcription factors would be predicted (as *FE/APL* is a floral activator). This could be an avenue of research in the future simultaneously pursued in *A. thaliana* and *M. truncatula*.

We also identified a *Mtftb2* mutant. *MtFTb* genes are hypothesized to act as floral activators as they are expressed in the leaves in a LD-responsive manner. Here the NF20803 *Mtftb2* mutant line flowered at a similar time to the wild type R108 plants grown alongside, albeit with a slight trend to lateness. Given the similarity of *MtFTb2* to *MtFTb1*, in both sequences (proteins are 94.3% identical) and pattern of expression (Laurie et al., 2011), functional redundancy between the two *MtFTb* genes cannot be excluded. The trend to lateness is consistent with this possibility and a double *Mtftb1 Mtftb2* mutant is required to test the hypothesis that *MtFTb* genes regulate flowering. Nonetheless, further evidence against this hypothesis is presented elsewhere in this study in the NF5076 *Mtfe* mutant, where the expression of both *MtFTb* genes is greatly reduced, if not lost (and *MtFTa1* is reduced). However, in this mutant the delay in flowering is only moderate and could be explained by the reduction in *MtFTa1* expression, suggesting that the disruption of both the *MtFTb* genes had little effect on flowering. This is consistent with previous flowering time observations when overexpression of *MtCDFd1_1* also disrupted expression of both *MtFTb* genes, but the late flowering observed was dependent on loss of *MtFTa1* function (Zhang et al., 2019).

Overall, these observations introduce a novel component, *MtFE*, into the current *M. truncatula* photoperiod pathway and more broadly advance our understanding of photoperiodic flowering time in *M. truncatula*.

## Data Availability Statement

The original contributions presented in the study are included in the article’s Supplementary Material, further inquiries can be directed to the corresponding authors.

## Author Contributions

GT and LZ performed the experiments. JW and KM provided the *M. truncatula Tnt1* insertion lines. GT and JP conceived the experiments, prepared the figures, and wrote the article with contributions from LZ, JW, and KM. All authors contributed to the article and approved the submitted version.

### Conflict of Interest

The authors declare that the research was conducted in the absence of any commercial or financial relationships that could be construed as a potential conflict of interest.

## Footnotes

^1^www.percival-scientific.com

^2^www.thermofisher.com

^3^www.qiagen.com
