# Supplementary material for: The Candidate Photoperiod Gene MtFE Promotes Growth and Flowering in Medicago truncatula
Source: Front Plant Sci. 2021 Mar 26;12:634091. doi: 10.3389/fpls.2021.634091 (PMC8032900; doi:10.3389/fpls.2021.634091)
Supplement: Supplementary file 1 [file Data_Sheet_1.pdf]

The candidate photoperiod gene MtFE promotes growth and flowering in *Medicago truncatula*

Geoffrey Thomson, Lulu Zhang, Jiangqi Wen, Kirankumar S. Mysore and Joanna Putterill

Supplementary Material

Table S1. Primers used in this study

Gene	Name	Sequence	Use
***AT1G79430* (*FE*)**	AT1G79430_295_F	TGGGTGTGAAGGGTCTTACTC	Cloning SUC2 expression vector
	AT1G79430_731_R	TGCGACTCATCATGCCAGAA	Cloning SUC2 expression vector
***Medtr6g444980* (*MtFE*)**	Medtr6g444980_10_F	TGTTCCCTCCAAAGAAGCCT	Genotyping NF8324 and NF9701
	Medtr6g444980_224_R108_R	AGGGTGAGTGAGAATAGAAAGAA	Genotyping NF8324 and NF9701
	Medtr6g444980_1062_F	GGGGGTGGAACTCTTTCAAT	Genotyping NF17758
	Medtr6g444980_1493_R	TTGCATGTATTTCCCTTGAGCT	Cloning SUC2 expression vector
	Medtr6g444980_1574_F	GGACCTCATGGAATGGCTGA	Genotyping NF5076, NF18291 and NF17742 as well as cloning SUC2 expression vector. Denoted 1F in figure 3.
	Medtr6g444980_1861_F	GGACTGCTTCATCATGCTTGG	qRT-PCR
	Medtr6g444980_1867_R108_R	AAGCATGATGAAGCAGTCCCT	Genotyping NF17758 and NF5076
	Medtr6g444980_1990_R	TCCCCTTGAAGTAAAGGCTTTGT	qRT-PCR and genotyping NF5076. Denoted 1R in figure 3.
	Medtr6g444980_2127_R	GTGAACCTCTACCTTGTGCCA	Genotyping NF18291 and NF17742
***Medtr7g084970* (*MtFTa1*)**	FTa1_forward	GTAGCAGTAGGAATCCACTAGC	qRT-PCR. (Laurie et al., 2011)
	FTa1_reverse	ACACTCACTCTCGGTTGATTTCC	qRT-PCR. (Laurie et al., 2011)
***Medtr7g085020* (*MtFTa2*)**	FTa2_forward	AAGTGGTAGCAGACCGAATC	qRT-PCR. (Laurie et al., 2011)
	FTa2_reverse	CACCACCATTGGTAACAGTC	qRT-PCR. (Laurie et al., 2011)
	Medtr7g085020_n115_F	ACGAATATCTCATCTCCCCTGC	Genotyping NF9421, NF9778 and NF19514

	Medtr7g085020_191_R	GCTGTTGGGTCATTTACGCC	Genotyping NF9421, NF9778 and NF19514
Medtr7g006630 **(*MtFTb1*)**	Medtr7g006630_0_R108_F	ATGAACCCTCTTGTGGTCTG	qRT-PCR
	Medtr7g006630_141_R108_R	TGGATTGACTATTTGGGAAG	qRT-PCR
	Medtr7g006630_864_R108_F	TCGTAGAAAAGAACTAATTACGGTG T	Genotyping NF0412, NF5116 and NF14653
	Medtr7g006630_1900_F	AGGCAACCTTGTAGGCACAC	Genotyping NF3253
	Medtr7g006630_1920_R	GTGTGCCTACAAGGTTGCCT	Genotyping NF0412, NF5116 and NF14653
	Medtr7g006630_2240_R	GAGAGCCCATATTGTACCAC	Genotyping NF3253
Medtr7g006690 **(*MtFTb2*)**	Medtr7g006690_20_F	TCCTCTTGTTGTTGGTGGTGT	qRT-PCR genotyping NF20803 and amplifying cDNA from NF20803
	Medtr7g006690_156_R108_R	TGAGTTGATTATTTGAGAGG	qRT-PCR and amplifying cDNA from NF20803
	Medtr7g006690_389_R108_R	GCTAGGACAAAGAAGGAATGGC	Genotyping NF20803
	Medtr7g006690_3347_F	TGGATTACCTGTTGCTGCTC	Amplifying cDNA in NF20803
	Medtr7g006690_3477_R	TGAAGGACAGGGATATATTTG	Amplifying cDNA in NF20803
Medtr0291s0010 **(*FTIP1-like*)**	Medtr0291s0010_102_F	GGCGGAACTGGTTGGCTATA	Genotyping NF3680 and NF10483
	Medtr0291s0010_791_R108_R	TTCGGTGTTTTAGTCGGACA	Genotyping NF3680 and NF10483
	Medtr0291s0010_1689_F	GGAAGAGCAGAACCACCTCT	qRT-PCR
	Medtr0291s0010_1851_R	CTTTTGTACCTCACCAAGCCA	qRT-PCR
Medtr7g100450 **(*NaKR1-like*)**	Medtr7g100450_591_R108_F	AGCTCCTCTGTTCCAAAGACT	qRT-PCR
	Medtr7g100450_798_R	TCCTGACCTTCCCTTCACAAC	qRT-PCR
AT5G15840 **(*CO*)**	AT5G15840_84_F	ACCGTGTATTGCCATGCAGA	Cloning Y2H vectors
	AT5G15840_636_R	TCTATCTCCCCCGTAGCTCG	Cloning Y2H vectors
AT5G47640 **(*AtNF-YB2*)**	AT5G47640_28 F	GAGGGCAAAACGGGAACAAC	Cloning Y2H vectors

	AT5G47640_371_R	AGTCCAGTCCTCTCCCCTTC	Cloning Y2H vectors
Medtr3g058980 **(*NF-YB-like*)**	Medtr3g058980_43_F	GATCAAACGCTCACGGAAGC	Cloning Y2H vectors
	Medtr3g058980_451_R	CACCACCACCATAACCACCA	Cloning Y2H vectors
Medtr5g095740 **(*NF-YB-like*)**	Medtr5g095740_58_F	CACCACGAGAACAAGACCGA	Cloning Y2H vectors
	Medtr5g095740_473_R	ACGTGTCCCTGATGCTGATG	Cloning Y2H vectors
Medtr1g082660 **(*NF-YC-like*)**	Medtr1g082660_95 F	TATGACTGGGTCACCTGGGA	Cloning Y2H vectors
	Medtr1g082660_531_R	GTTCCTGTGATTGCTGCTGC	Cloning Y2H vectors
Medtr3g099180 **(*NF-YC-like*)**	Medtr3g099180_37 F	CTTCACCCACGGCAGTTACA	Cloning Y2H vectors
	Medtr3g099180_497 R	TCATTCCAGCAGGTTGTCCC	Cloning Y2H vectors
Medtr7g113680 **(*NF-YC-like*)**	Medtr7g113680_39_F	GTTGTCGGTAGTGGCAGTCA	Cloning Y2H vectors
	Medtr7g113680_443_R	TCAGTGTGGTTCCATGAGCG	Cloning Y2H vectors
**Miscellaneous**	G775	GACTCGAGTCGACATCGA(T)17	First strand cDNA synthesis
	PP2A_F	GTGTTTTGCTTCCGCCGTT	qRT-PCR. (Kakar et al., 2008)
	PP2A_R	CCAAATCTTGCTCCCTCATCTG	qRT-PCR. (Kakar et al., 2008)
	Tnt1_F	ACAGTGCTACCTCCTCTGGATG	Genotyping Tnt-1 lines. Denoted 2F in figure 3. (Laurie et al., 2011)
	Tnt1_R	CAGTGAACGAGCAGAACCTGTG	Genotyping Tnt-1 lines. (Laurie et al., 2011)
	pSAK778_SUC2_SUC2_854_F	CCTCCACCACTACAACCACC	Cloning SUC2 expression vectors
	pSAK778_SUC2_OCS_623_F	ATGCGATCATAGGCGTCTCG	Cloning SUC2 expression vectors
	M13_F	GTAAAACGACGGCCAG	Cloning Y2H vectors
	pDONOR221_attB_n106_F	TCTTGTGCAATGTAACATCAGAG	Cloning Y2H vectors
	pDEST22_GAL4_AD_F	AATACCACTACAATGGAT	Cloning Y2H vectors

	pDEST32_GAL4_BD_F	GAGTAGTAACAAAGGTCAA	Cloning Y2H vectors
	pDEST22/32_R	CGAAGAAGTCCAAAGCTCCA	Cloning Y2H vectors

Table S2. Mutant *M. truncatula* lines from the *Tnt1* retrotransposon population

Gene Name	Gene identifier	Tnt1 line	Tnt1 position	VLD	LD	VSD	SD	Disrupts expression
MtFTa2	*Medtr7g085020*	NF9421	Promoter	WT	N. D.	WT*	WT	No
		NF9778	Not identified	N. D.	N. D.	N. D.	N. D.	N. D.
		NF19514	Promoter	WT	N. D.	WT	N. D.	No
MtFTb1	*Medtr7g006630*	NF0412	Not identified	N. D.	N. D.	N. D.	N. D.	N. D.
		NF3253	Not identified	N. D.	N. D.	N. D.	N. D.	N. D.
		NF5116	Not identified	N. D.	N. D.	N. D.	N. D.	N. D.
		NF14653	Not identified	N. D.	N. D.	N. D.	N. D.	N. D.
MtFTb2	*Medtr7g006690*	NF17918	Not identified	N. D.	N. D.	N. D.	N. D.	N. D.
		NF20803	Exon 1	WT	Sl. late	N. D.	N. D.	Yes
MtFE	*Medtr6g444980*	NF5076	Exon 6	Late	Late	Sl. late	WT	Yes
		NF8324	Not identified	N. D.	N. D.	N. D.	N. D.	N. D.
		NF9701	Not identified	N. D.	N. D.	N. D.	N. D.	N. D.
		NF17742	Not identified	N. D.	N. D.	N. D.	N. D.	N. D.
		NF17758	Not identified	N. D.	N. D.	N. D.	N. D.	N. D.
		NF18291**	Exon 6	N. D.	N. D.	N. D.	N. D.	N. D.
MtFTIP1	*Medtr0291s0010*	NF3680	Not identified	N. D.	N. D.	N. D.	N. D.	N. D.
		NF10483	Exon	WT	WT	WT*	WT	No

WT - Wild type like; Sl late - slightly late; N. D. - Not done.
Flowering time reflects the data from both this initial generation and subsequent generations if pursued.
Not identified indicates that the predicted *Tnt1* insertion in the plant lines was not recovered in the plants grown.
* This population flowered very slightly early but growth of a segregating population demonstrated that this was not attributable to the Tnt1 insertion.
** Seedling lethal phenotype

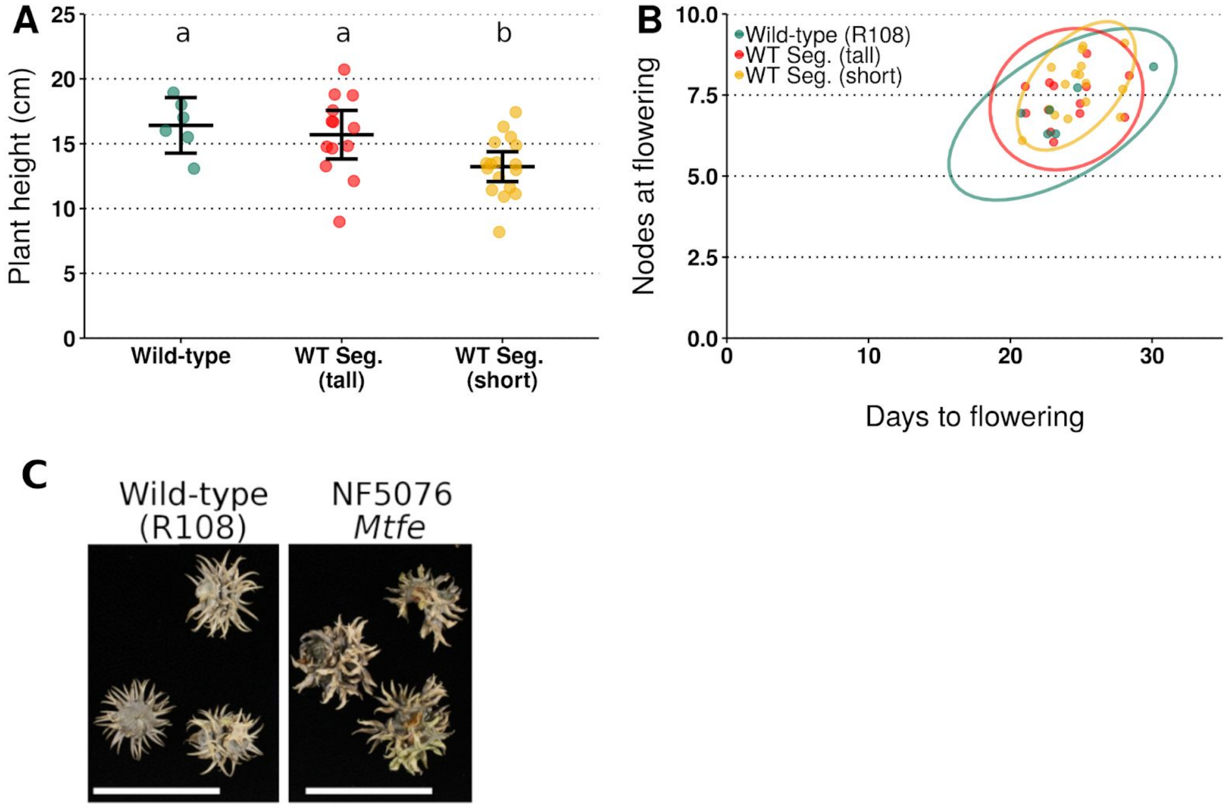

Figure S1. NF5076 wild type segregants are smaller relative to the wild type R108 plants but flower at the same time as wild type. (**A**) plots the heights of 35 day old progeny of NF5076 wild type segregants whose parents were either ”tall” (wild type-like) or ”short” (see Figure 3B panel 2) (n= 13 and 17 respectively) along with wild type R108 plants (n = 6), all grown in VLD. Each point represents an individual plant. Black bars depict the mean bounded by 95% confidence intervals. Letters represent the results of a one way ANOVA (type III sums of squares) such that each letter is significantly different from one another. (**B**) is the flowering times of these plants. Flowering time graphs are plots of days to flower against nodes on the primary axis at the time of flowering. Variation is indicated by 95% confidence ellipses. (**C**) is the malformed barrel phenotype observed in the background of the NF5076 line. All scale bars are 5cm.

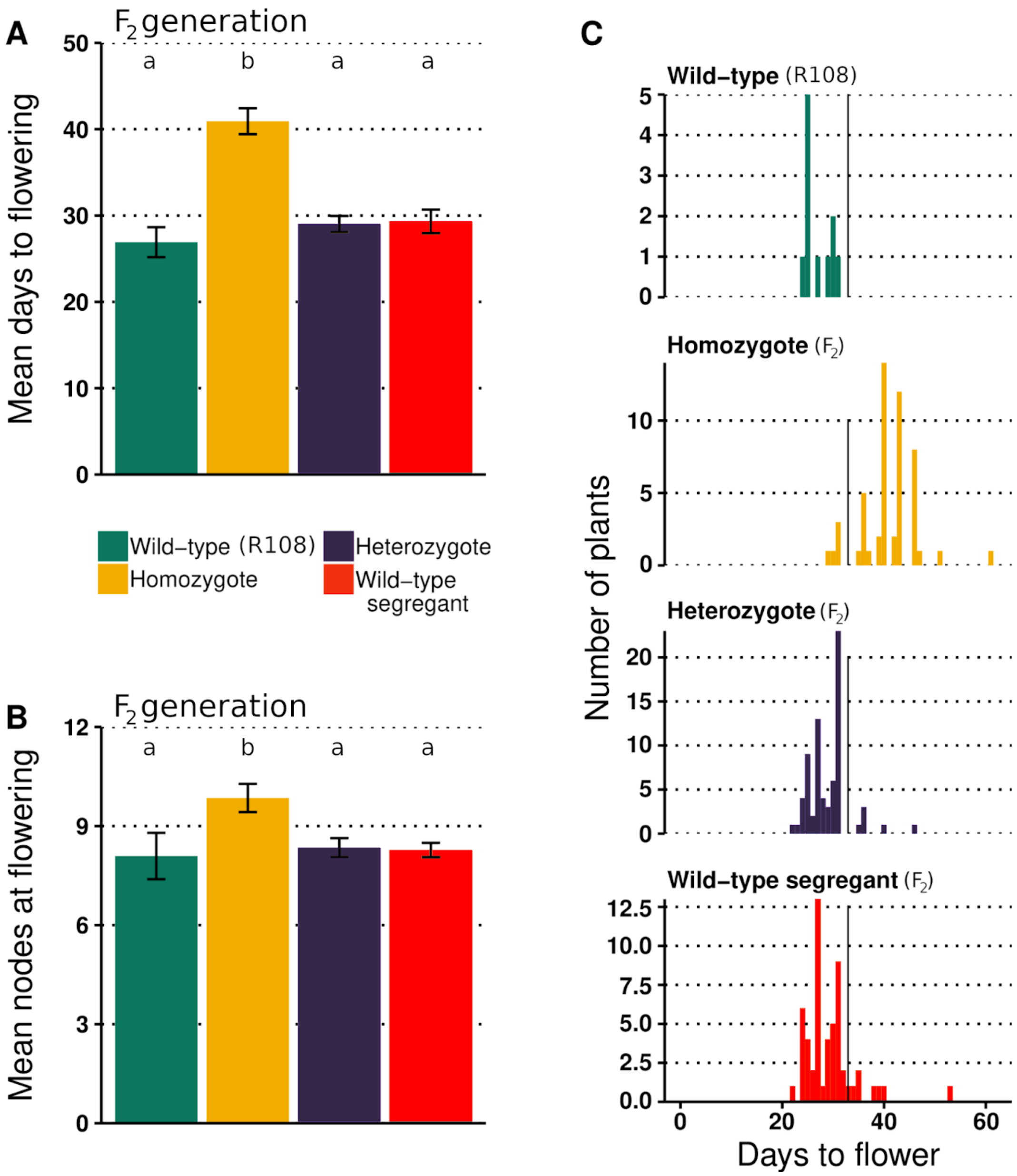

Figure S2. The NF5076 *Mtfe* insertion is strongly linked to the late flowering phenotype. Flowering time of a F_2 NF5076 linkage population (n = 180) grown in VLD. (**A)** depicts the average days to flowering for plants across the different genotypes with wild type R108 grown alongside and (**B**) depicts the nodes at flowering. In both cases error bars are 95% confidence intervals. Different letters indicate significantly different results via a one way ANOVA (type III sums of squares; α = 0.05). (**C**) demonstrates the spread of the days to flowering in histograms where the overlap between homozygotes, heterozygotes and wild type segregants can be seen. A vertical line is drawn at 33 days by which time all wild type R108 plants had flowered.

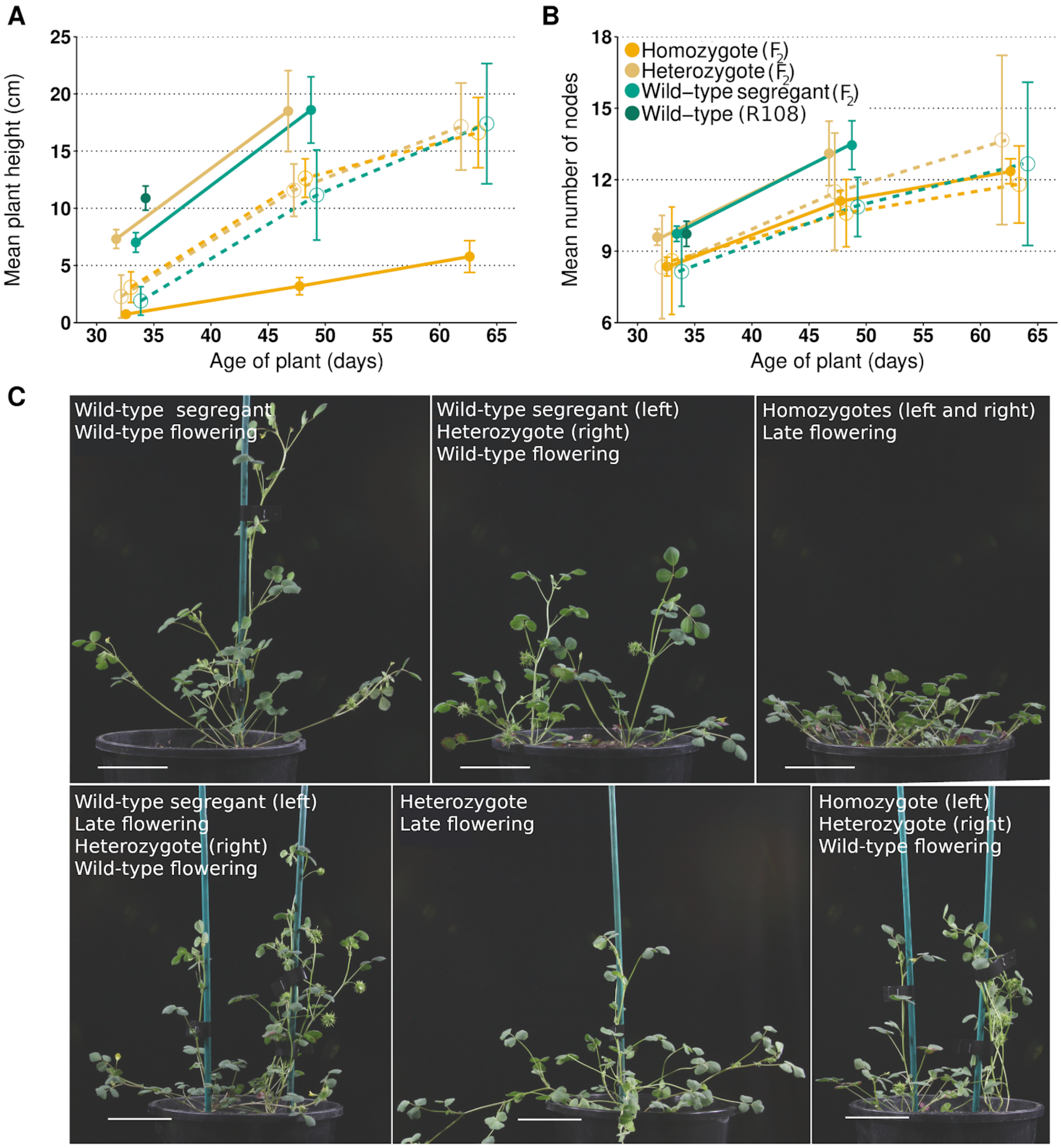

Figure S3. Plant architecture in the NF5076 *Mtfe* F_2 segregating population. (**A**) is the mean height (n = 180) of the plants measured at 33, 48 and 63 days. Measurements of heterozygotes and wild type segregants measured at 48 days are a sample (n = 20) of the total population. Dotted lines are the growth measurements of either *Mtfe* homozygous plants which flowered earlier than 33 days (5 plants), or wild type segregants or heterozygotes which flowered later than 33 days (8 and 6 plants respectively) (see Figure S2C). Error bars are 95% confidence intervals. (**B**) is the number of nodes on the primary axis at the time of measurement. Error bars are 95% confidence intervals. (**C**) is photos of representative 47 day old plants with their genotypes and flowering times annotated. Scale bars are 5cm.

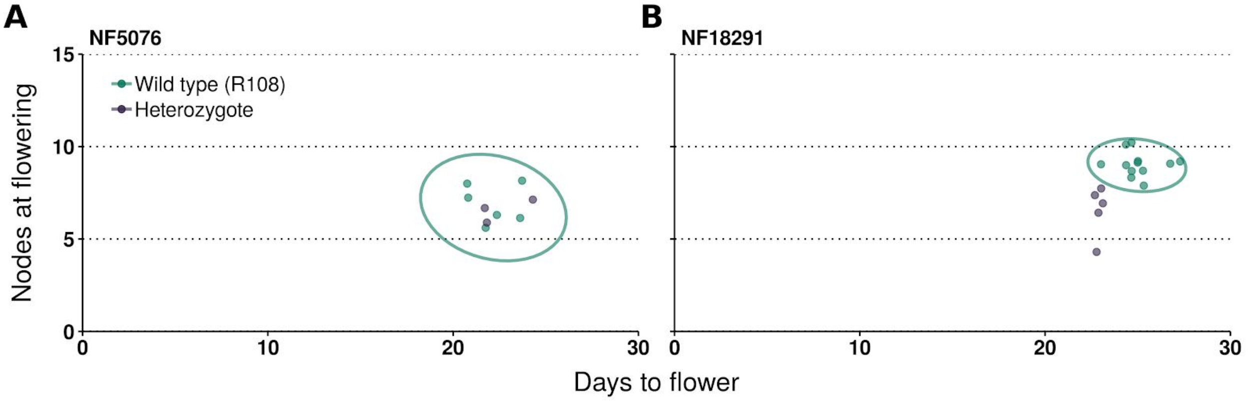

Figure S4. *Mtfe* heterozygous plants from the NF5076 and NF18291 lines flower like wild type. Flowering time of *Mtfe* heterozygotes from the (**A**) NF5076 (n = 3-12) and (**B**) NF18291 (n = 5-12) lines. Flowering time graphs are plots of days to flower against nodes on the primary axis at the time of flowering. Each point represents an individual plant. Variation is indicated by 95% confidence ellipses. Note that there are no ellipses if there is insufficient variation.

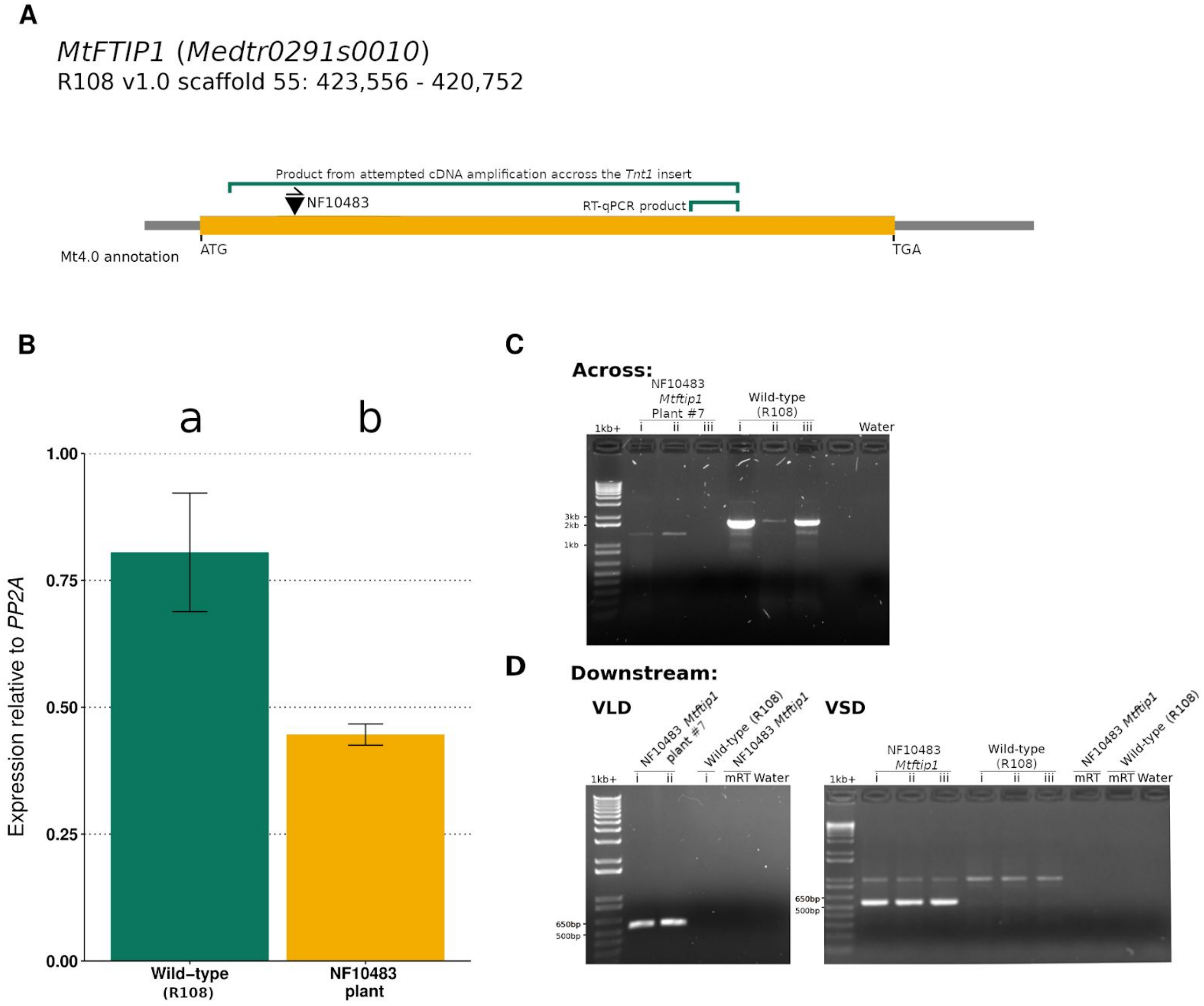

Figure S5. NF10483 *Mtftip1* homozygous plants produce transcript downstream of the *Tnt1* insertion. (**A**) is a schematic of the *MtFTIP1* locus with the orange rectangle representing the exon of the single exon gene. The inverted black triangle indicates the location of the confirmed *Tnt1* insertion and arrows above it denote the orientation of the insertion. The green brackets indicate the PCR products amplified in the following subfigures. (**B**) plots the mean expression (n = three independent biological replicates) of *MtFTIP1* downstream of the *Tnt1* insertion in a single NF10483 *Mtftip1* plant. The plant was grown in VLD and sampled when 44 days old at ZT4. Error bars depict standard errors. Different letters indicate significantly different results from post-hoc Tukey-adjusted comparisons of a linear model (α = 0.05). (**C**) is an electrophoresis gel displaying how amplification across the insert in cDNA does not occur. (**D**) is the amplification of downstream transcripts using the Tnt1_F2 / Medtr0291s0010_791_R108_R primer pair. Downstream transcripts were also amplified in cDNA extracted from plants grown in VSD. In VSD the samples were taken from separate plants at ZT4 in triplicate when plants were 18 days old.

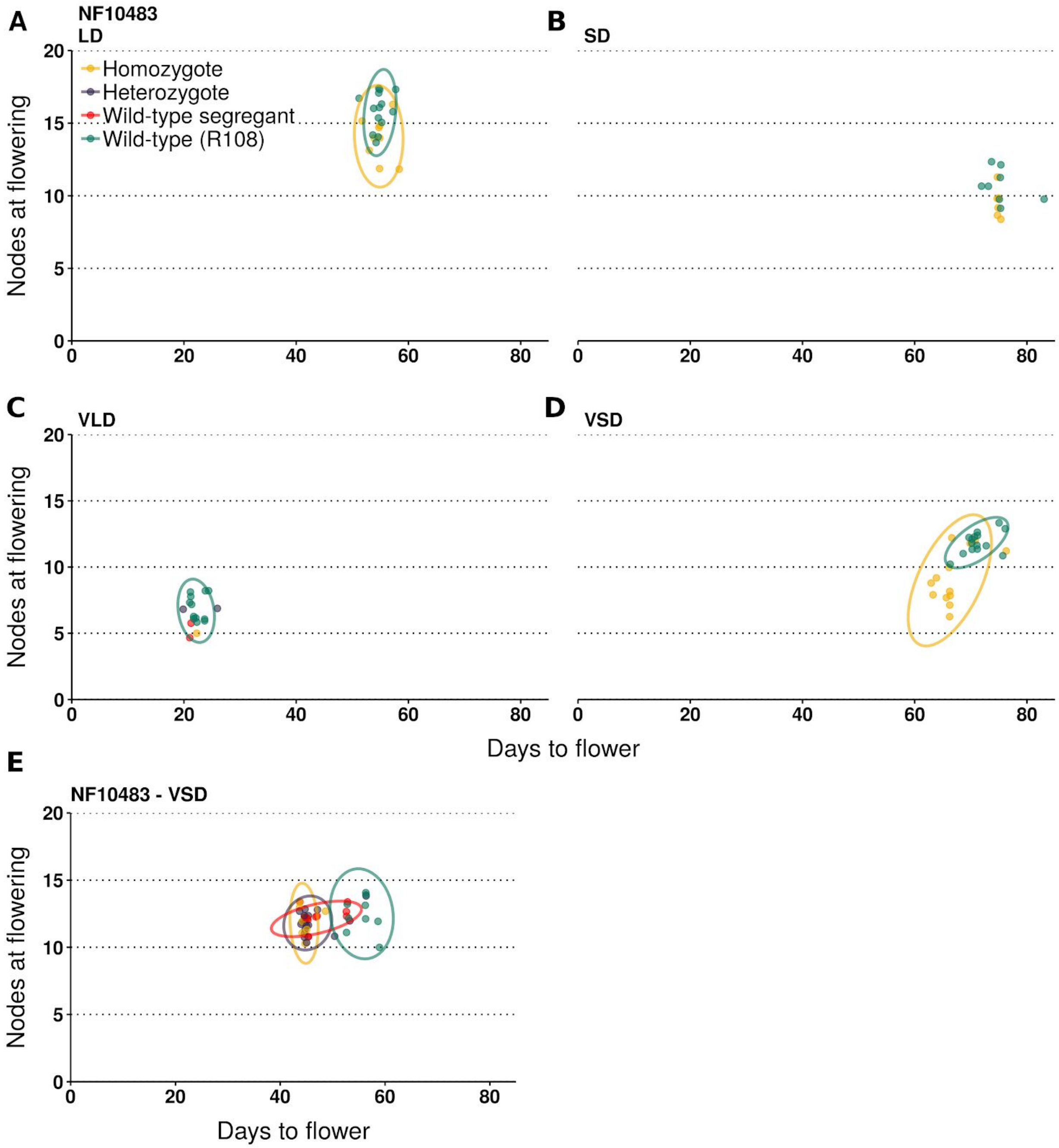

Figure S6. Flowering time of plants from the NF10483 *Mtftip1* homozygous line flower like wild type R108. NF10483 *Mtftip1* plants (n = 5-15) grown in contrasting conditions. (**A**) LD, (**B**) SD, (**C**) VLD and (**D**) VSD. (**E**) is a population of NF10483 plants segregating for the insertion in *MtFTIP1* grown in VSD (n = 9-37). Flowering time graphs are the plotting of the days to flower against the nodes on the primary axis at the time of flowering with 95% confidence ellipses. Each point represents an individual plant. Note that there are no ellipses if there is insufficient variation.

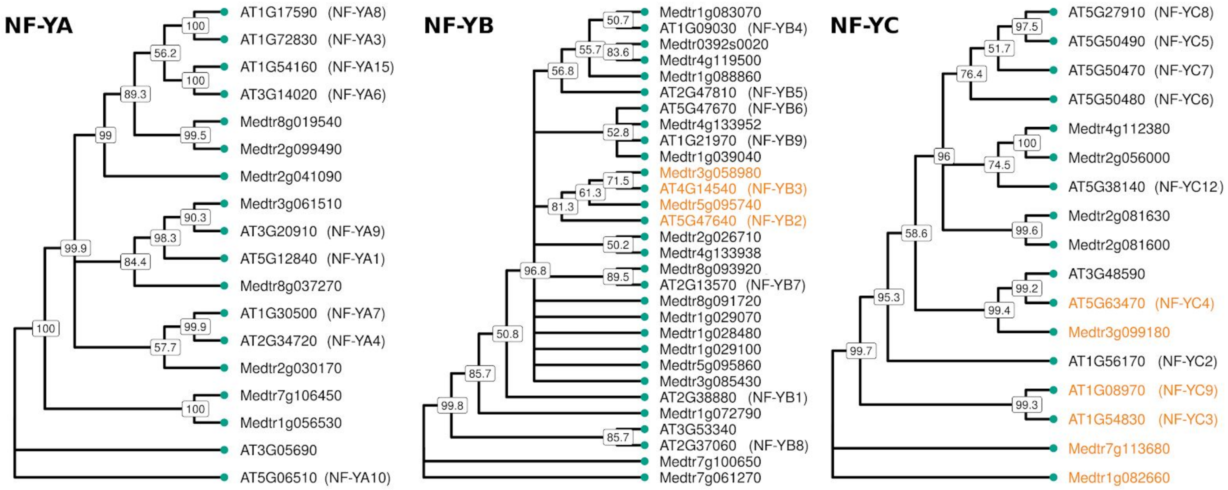

Figure S7. Neighbour joining trees for NF-Y-like proteins in both *A. thaliana* and *M. truncatula*. Amino acid sequences were aligned using MUSCLE (Edgar, 2004) and a neighbour-joining tree using a JC69 substitution model constructed with 1,000 bootstraps. Highlighted in orange are either *A. thaliana* genes demonstrated to participate in flowering time (Kumimoto et al., 2008, 2010) or *M. truncatula* genes used in the Y2H assay. Note that sequences from Medtr4g119670, Medtr5g019130, Medtr5g078780 and Medtr5g078810 were omitted as despite having some similarity to NF-Y-like genes, they did not align well.

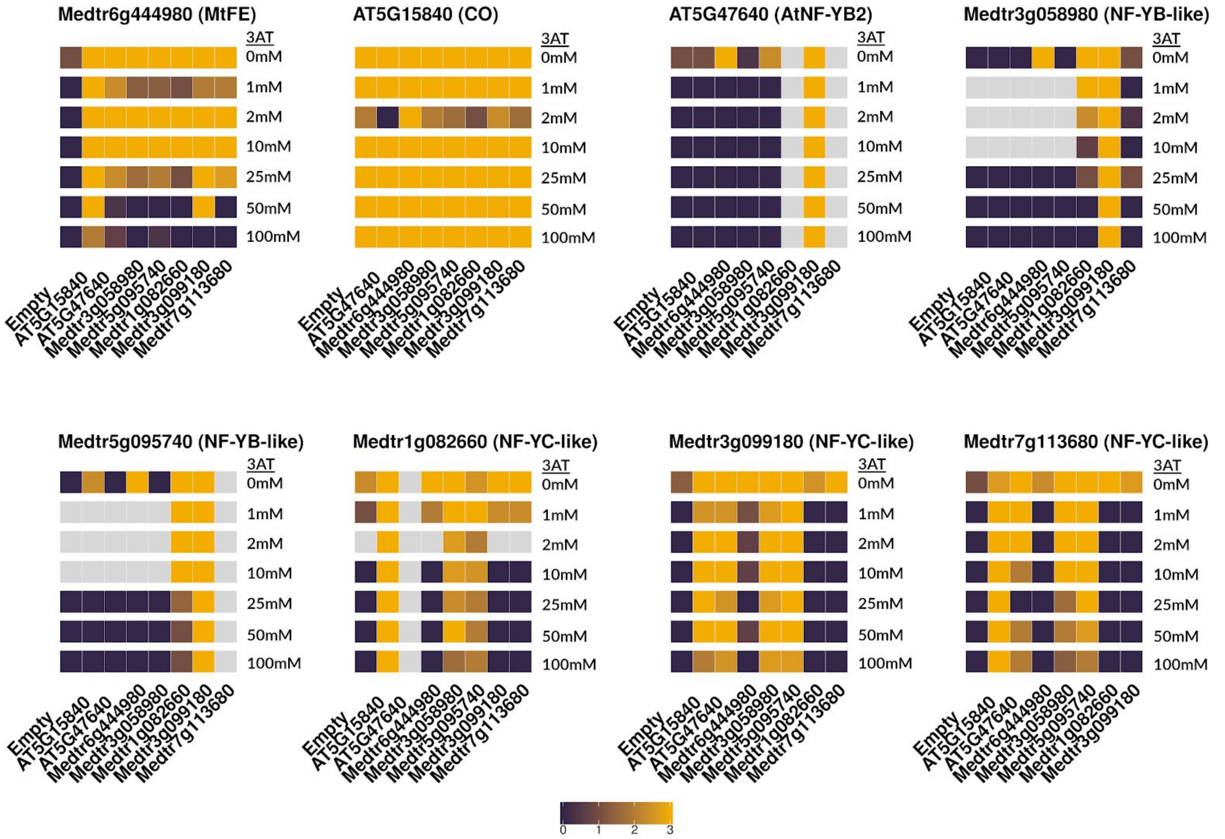

Figure S8. MtFE appears to interact with NF-Y-like proteins in Y2H assays. This chart summarises the Y2H interaction results after 10 days of growth. Each heatmap has the name of the BD fused protein above and each column has the name of the AD fused protein. Rows are increasing concentrations of 3AT and colour intensities are the mean growth scoring of three technical replicates. They range from purple (0 - no growth) to yellow (3 - strong growth). Note that when CO is fused to the BD, growth occurs in the absence of a AD fused protein indicating auto-activation. Grey columns represent failed matings and grey rows plates with low levels of background contaminations such that weak interactions were omitted. Proteins assayed were Medtr6g444980 (MtFE), AT5G15840 (CO), AT5G47640 (AtNF-YB2), Medtr3g058980 (NF-YB-like), Medtr5g095740 (NF-YB-like), Medtr1g082660 (NF-YC-like), Medtr3g099180 (NF-YC-like) and Medtr7g113680 (NF-YC-like).

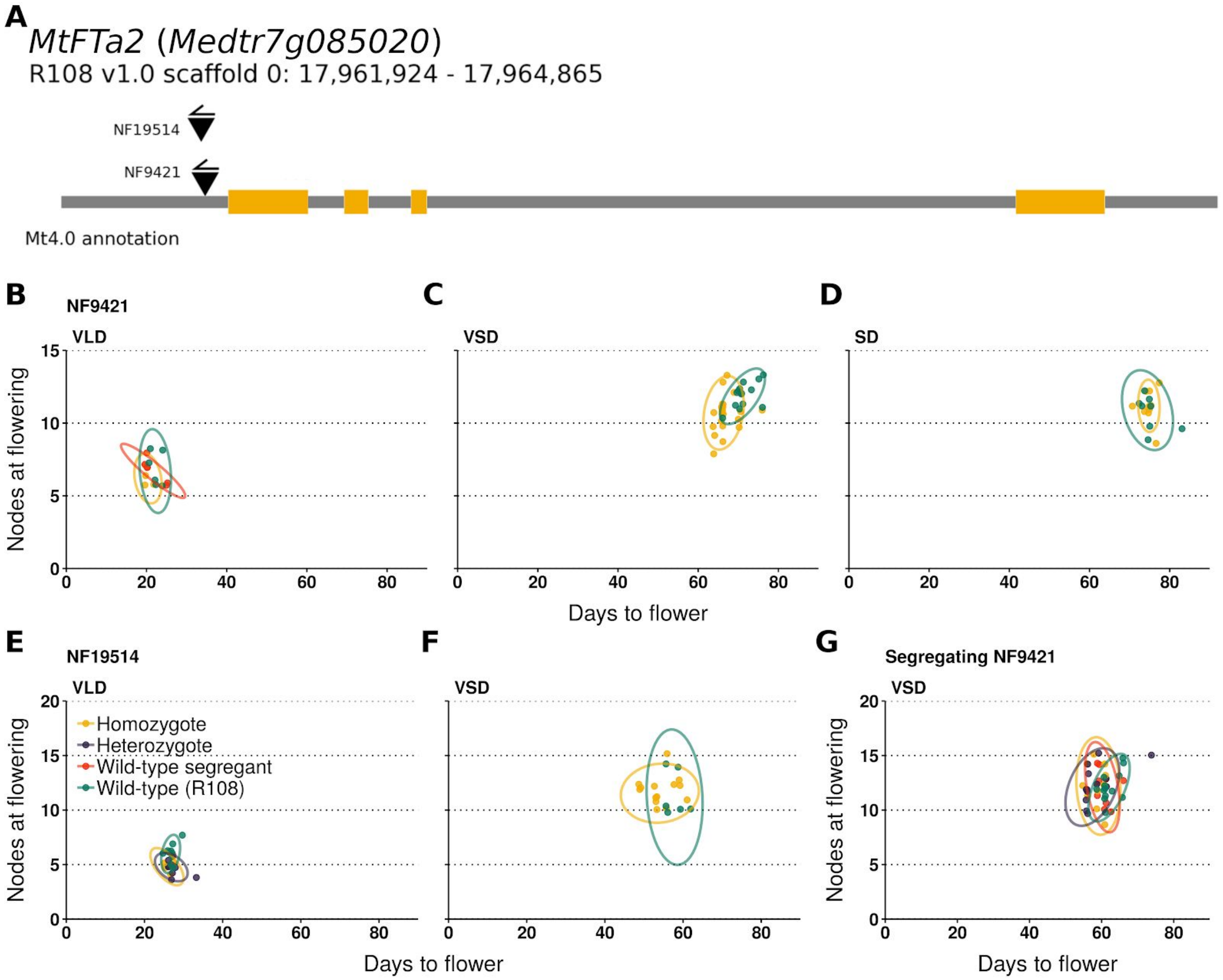

Figure S9. NF9421 *Mtfta2* and NF19514 *Mtfta2* lines flower like wild type plants. (**A**) is a schematic of the *MtFTa2* locus with the exons depicted in orange. The inverted black triangle indicates the location of the NF9421 and NF19514 *Tnt1* insertions and the arrow above it denotes the orientation of the insertion. The flowering time of *M. truncatula* NF9421 *Mtfta2* mutants (n = 6-22) grown in (**B**) VLD, (**C**) VSD and (**D**) SD are plotted below followed by NF19514 *Mtfta2* (n = 6-15) grown in (**E**) VLD and (**F**) VSD. In (**G**) the flowering time of a F_2 generation segregating population (n = 10-13) from a NF9421 *Mtfta2* x wild type R108 backcross is plotted. Flowering time graphs are plots of days to flower against nodes on the primary axis at the time of flowering. Each point represents an individual plant. Variation is indicated by 95% confidence ellipses.

Supplementary References

Edgar, R. C. (2004). MUSCLE: a multiple sequence alignment method with reduced time and space complexity. *BMC Bioinformatics* 5, 113. doi:10.1186/1471-2105-5-113.

Kumimoto, R. W., Adam, L., Hymus, G. J., Repetti, P. P., Reuber, T. L., Marion, C. M., Hempel, F. D., and Ratcliffe, O. J. (2008). The Nuclear Factor Y subunits NF-YB2 and NF-YB3 play additive roles in the promotion of flowering by inductive long-day photoperiods in Arabidopsis. *Planta* 228, 709–723. doi:10.1007/s00425-008-0773-6.

Kumimoto, R. W., Zhang, Y., Siefers, N., and Holt, B. F. (2010). NF-YC3, NF-YC4 and NF-YC9 are required for CONSTANS-mediated, photoperiod-dependent flowering in Arabidopsis thaliana. *Plant J.* 63, 379–391. doi:10.1111/j.1365-313X.2010.04247.x.
